# Plastic architecture of bacterial genome revealed by comparative genomics of *Photorhabdus *variants

**DOI:** 10.1186/gb-2008-9-7-r117

**Published:** 2008-07-22

**Authors:** Sophie Gaudriault, Sylvie Pages, Anne Lanois, Christine Laroui, Corinne Teyssier, Estelle Jumas-Bilak, Alain Givaudan

**Affiliations:** 1 INRA, UMR 1133, Laboratoire EMIP, Place Eugène Bataillon, F-34095 Montpellier, France; 2 Université Montpellier 2, UMR 1133, Laboratoire EMIP, Place Eugène Bataillon, F-34095 Montpellier, France; 3 Université Montpellier 1, EA 3755, Laboratoire de Bactériologie-Virologie, 15, Avenue Charles Flahault, BP 14491, F-34060 Montpellier Cedex 5, France

## Abstract

**Background:**

The phenotypic consequences of large genomic architecture modifications within a clonal bacterial population are rarely evaluated because of the difficulties associated with using molecular approaches in a mixed population. Bacterial variants frequently arise among *Photorhabdus luminescens*, a nematode-symbiotic and insect-pathogenic bacterium. We therefore studied genome plasticity within *Photorhabdus *variants.

**Results:**

We used a combination of macrorestriction and DNA microarray experiments to perform a comparative genomic study of different *P. luminescens *TT01 variants. Prolonged culturing of TT01 strain and a genomic variant, collected from the laboratory-maintained symbiotic nematode, generated bacterial lineages composed of primary and secondary phenotypic variants and colonial variants. The primary phenotypic variants exhibit several characteristics that are absent from the secondary forms. We identify substantial plasticity of the genome architecture of some variants, mediated mainly by deletions in the 'flexible' gene pool of the TT01 reference genome and also by genomic amplification. We show that the primary or secondary phenotypic variant status is independent from global genomic architecture and that the bacterial lineages are genomic lineages. We focused on two unusual genomic changes: a deletion at a new recombination hotspot composed of long approximate repeats; and a 275 kilobase single block duplication belonging to a new class of genomic duplications.

**Conclusion:**

Our findings demonstrate that major genomic variations occur in *Photorhabdus *clonal populations. The phenotypic consequences of these genomic changes are cryptic. This study provides insight into the field of bacterial genome architecture and further elucidates the role played by clonal genomic variation in bacterial genome evolution.

## Background

Comparative genomics, in the study of different bacterial genera, species, and strains, leads to the definition of two DNA pools in bacterial genomes: a set of genes shared by all genomes in a taxa, namely the 'core' genome; and a set of genes containing mobile and accessory genetic elements, termed the 'flexible' gene pool. Both intergenomic and intragenomic rearrangements occur in this 'flexible' gene pool [1]. Changes in the 'flexible' gene pool are considered to be the motor of bacterial diversification and evolution [2-4].

However, comparative genomic analyses of genomic variants within a clonal population are rarely undertaken because of the difficulties involved in using molecular approaches in a mixed population. Initially, researchers focused on local modifications of the DNA sequence occurring during phase variation. Phase variation is an adaptive process by which certain bacteria within a bacterial subpopulation, called phase variants, undergo frequent and reversible phenotypic changes. Phase variation is dependent on DNA sequence plasticity, generating a reversible switch between 'on' and 'off' phases of expression for one or more protein-encoding genes. Variation in the expression of certain genes in some phase variants allows the bacterial population to adapt to environmental change [5-7]. Other studies have focused on DNA sequence variations that involve large regions of the genome in a clonal population. These extensively distributed and large genomic rearrangements mostly occur through homologous recombination between repeated sequences such as *rrn *loci, duplicated genes, or insertion sequences, which may then lead to the inversion, amplification, or deletion of chromosomal fragments. These events can occur either under strong selective pressure - such as *in vitro *antibiotic selection [8], stressful high temperature [9], long-term storage [10-12], and chronic clinical carriage [13] - or without specific selective pressure [14-20].

The phenotypic consequences of such large rearrangements are variable. In *Streptomyces *spp., genetic instability affects various phenotypical properties, including morphological differentiation, production of secondary metabolites, antibiotic resistance, secretion of extracellular enzymes, and gene expression for primary metabolism, regardless of selective pressure [20]. In other bacterial species and when stressful selective pressure is applied, large-scale genomic variation often correlates with modification of certain phenotypes: reversion from nutritional auxotrophy to prototrophy [10], variation in colony morphology [11], modification of bacterial growth features [12], and adaptation to high temperature [9]. Few data are available on phenotypic variation in the absence of strong selective pressure. A few studies suggest that large genomic architecture modifications can occur with or without slight detectable phenotypic modifications [15,16]. We studied genomic rearrangements in the entomopathogenic bacterium *Photorhabdus luminescens*, for which variants are frequently observed in standard growth conditions, in order to investigate further the link between genomic variation within a bacterial population and the phenotypic consequences.

*P. luminescens *is a member of the Enterobacteriaceae; it is a symbiont of entomopathogenic nematodes and is pathogenic for a wide variety of insects [21-24]. Bacterial variants frequently arise within the *Photorhabdus *genus. Two types of variant exist. The phenotypic variants (PVs) are the most studied. The primary PV is characterized by the presence of numerous phenotypic traits (production of extracellular enzymes, pigments, antibiotics, crystalline inclusion bodies, and ability to generate bioluminescence) that are absent from the secondary PV. Secondary PVs are mostly obtained during prolonged *in vitro *culturing [25,26]. Only primary PVs support nematode growth and development both in the insect cadaver and *in vitro*. However, both variants are equally virulent to insect hosts [27]. This phenomenon differs from classical phase variation because it occurs at low and unpredictable frequency, it is rarely reversible, and numerous phenotypic traits are altered simultaneously [27]. Recent studies suggest that generation of PVs in *P. luminescens *may be controlled by several regulatory cascades, each of them involving the products of many different genes [28-31].

The other common variants in *Photorhabdus *are colonial variants (CVs). Different colonial morphotypes can be generated from one colony subculture. This variation is unstable; indeed, each morphotype can generate all other morphotypes [32-36]. The most frequent CVs are small-colony variants (SCVs). These SCVs constitute a slow-growing bacterial subpopulation with atypical colony morphology and unusual biochemical characteristics that, in the case of clinical isolates, cause latent or recurrent infections [37]. In *Photorhabdus*, these SCVs can be generated from primary or secondary PV [34]. SCVs have small cells, do not produce crystalline inclusions [32-34], and have undergone changes in their proteome [33,34]. Some SCVs have modified virulence properties and do not support nematode development and reproduction [32].

Previous studies, incorporating local genetic [28,38,39] or nonexhaustive genomic comparisons [33,34,40,41], have not identified genomic differences within sets of PVs or CVs. We used the recently elucidated complete nucleotide sequence of the *P. luminescens *subspecies *laumondii *strain TT01 [42] to study systematically the link between phenotypic and genomic variations in clonal *Photorhabdus *variants. We undertook whole-genome comparisons between the wild-type TT01 strain and six different PVs or CVs. We showed that large genomic rearrangements occurred *in vivo *and *in vitro*. We described two categories of intragenomic rearrangements: deletion events occurring in the 'flexible gene pool', and an unusual duplication of a 275-kilobase (kb) region, encompassing 4.8% of the TT01 wild-type genome. These rearrangements were not correlated with the generation of PVs, and we did not detect a functional relationship between the genes affected by rearrangements and phenotypic variation. Thus, the consequences of these genomic changes are cryptic.

## Results

### TT01α_/I_: a genomic variant isolated from the laboratory-maintained nematode *Heterorhabditis bacteriophora*

**Figure 1:**
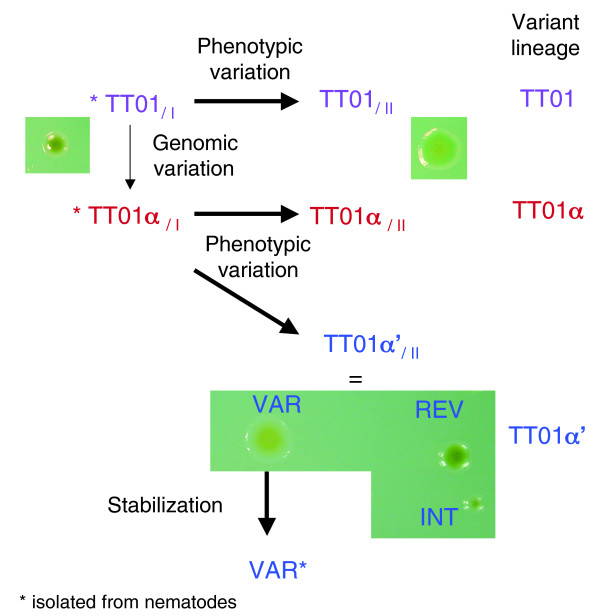
Schematic representation of TT01 variants selection on TreGNO medium. TT01_/I_, TT01α_/I_, and REV colonies are green, convex, and mucoid colonies; TT01_/II_, TT01α_/II_, VAR, and VAR* colonies are yellow, flat, and nonmucoid; and the INT colonies are small, green, convex, and mucoid.

The nematode *Heterorhabditis bacteriophora *TH01, harboring the TT01 wild-type strain, was collected in Trinidad in 1993 [43]. The nematode was maintained in the laboratory and multiplied by infestation in the Lepidopteran *Galleria mellonella *[44]. In 1998, a further bacterial isolate was taken from this nematode. During the course of a genetic study of the type III secretion system, we discovered that the bacterium isolated in 1998 is a genomic variant. It differs from the TT01 wild-type strain by a 250 base pair deletion at the 5' end of the gene *lopT1 *(Additional data file 1). This gene encodes a type III secretion system effector that appears to be involved in the depression of the insect innate immune system [45]. Both TT01 wild-type and the *lopT1 *genomic variant produced many of the phenotypes associated with primary PVs, including bioluminescence, lipase activity, antibiotic production, and presence of cytoplasmic crystal (Table 1). Therefore, both were primary PVs. To distinguish between them, the TT01 wild-type strain was named TT01_/I _and the *lopT1 *genomic variant, TT01α_/I _(Figure 1).

**Table 1:** Phenotypes of *P. luminescens *TT01_/I_, TT01α_/I_, and their respective variants

Phenotype	TT01_/I_	TT01α_/I_	TT01_/II_	TT01α_/II_	TT01α'_/II_	VAR	VAR*	REV	INT
Bioluminescence	+	+	-	-	-	-	-	+/w	w
Colony morphology	Convex, mucoid,	Convex, mucoid,	Flat, nonmucoid	Flat, nonmucoid	Flat, nonmucoid	Flat, nonmucoid	Flat, nonmucoid	Convex, mucoid	Small, convex, mucoid
Lipase activity on Tween 20-60	++	++	+	+	+	+	+	++	ND
Lipase activity on Tween 80-85	++	++	+/w	+/w	+v	+	v	++	ND
Pigmentation	+(Orange)	+(Orange)	+(Yellow)	++(Yellow)	-	-	-	+(Orange)	ND
Antibiotic production	+	+	-	-	-	-	-	+/w	ND
Crystal proteins	+	+	-	-	-	-	-	w	-
Coloration on TreGNO medium	Green	Green	Yellow	Yellow	Yellow	Yellow	Yellow	Green	Green

+, positive; -, negative; v, variable; w, weak.

### Isolation and characterization of PVs and CVs from TT01_/I _and TT01α_/I_

We cultured TT01_/I _and TT01α_/I _in liquid broth and selected primary and secondary PVs on NBTA (nutrient agar supplemented with bromothymol blue and triphenyl 2,3,5 tetrazolium chloride) plates. TT01_/II _secondary PV was derived from TT01_/I _(TT01 lineage; Figure 1). TT01α_/II _and TT01α'_/II _secondary PVs were obtained from TT01α_/I _(TT01α lineage; Figure 1). TT01_/II_, TT01α_/II_, and TT01α'_/II _had classic secondary PV traits (Table 1).

We developed a new agar medium, the TreGNO (nutrient agar with trehalose and and bromothymol blue) medium, for color discrimination of TT01 PVs (see Materials and methods [below] for details). PVs produce green, convex, and mucoid colonies whereas secondary PVs produce yellow, flat, and nonmucoid colonies on this medium. TT01_/II _and TT01α_/II _colonies were homogeneous and had the colonial traits of secondary PVs. However, TT01α'_/II _was composed of three CVs (TT01α' lineage; Figure 1). The first was a primary colonial form (green, convex, and mucoid colonies), named REV because it resembled a revertant colony, exhibiting primary PV traits (although bioluminescence, pigmentation, and crystal production were not completely restored; Table 1). The second was a secondary colonial form (yellow, flat, and nonmucoid colonies), named VAR because of its secondary PV traits (Table 1). The third form had small, green, convex, and mucoid colonies, and was named INT because of its intermediate traits or traits from both the primary and secondary PVs (Table 1). These CVs are unstable because each individual TT01α'_/II _colony grown in liquid broth gives rise to a mixture of the three colonial forms on TreGNO medium. We generated a stable secondary PV from the VAR colonial variant by plating a liquid subculture from an individual VAR colony on nutrient agar and picking another VAR colony for a new cycle of liquid/plate culture. We continued this enrichment process until the liquid subculture generated 95% of VAR colonies on TreGNO plates. The stable population was named VAR* (Figure 1).

We PCR-amplified the *lopT1 *5' region from TT01_/II_, TT01α_/II_, TT01α'_/II_, VAR*, and REV (Additional data file 1). The *lopT1 *deletion was only present in the TT01α and TT01α' lineages.

### Virulence of TT01 variants

**Figure 2:**
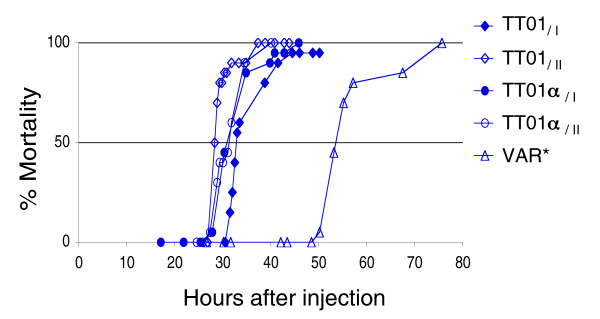
Mortality in *Spodoptera littoralis*. Shown is the mortality in *S. littoralis *infected with the TT01_/I _*Photorhabdus luminescens *wild-type strain, the genomic variant TT01α_/I_, the secondary variants TT01_/II _and TT01α_/II_, and the stabilized VAR* colonial variant. Bacteria obtained at the end of the exponential phase were injected into fourth-instar larvae. Mortality values are based on data obtained after injection into 20 larvae. All experiments were repeated at least twice.

We injected TT01_/I_, TT01_/II_, TT01α_/I_, TT01α_/II_, and VAR* into *Spodoptera littoralis *larvae to evaluate the pathogenicity of these variants in insect larvae. TT01_/II_, TT01α_/I_, and TT01α_/II _had the same level of pathogenicity as TT01_/I_; 50% mortality (LT_50_) was reached between 28 and 32 hours after injection for the TT01 wild-type strain and these three variants. By contrast, VAR* had a delayed LT_50 _of 53 hours, although 100% mortality was reached at 3 days after infection (Figure 2).

### Extensive rearrangements in genomic architecture correlated with the variant lineages

**Figure 3:**
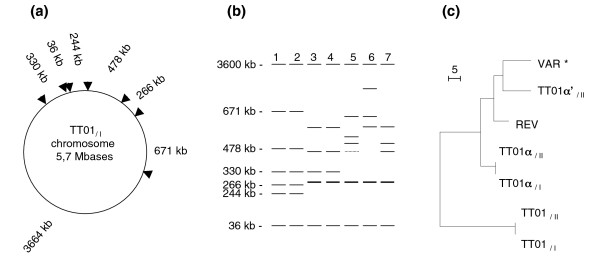
Variation in genomic architecture of the TT01 variants. **(a) **Schematic representation of the I-*Ceu*I restriction map of the TT01_/I_* Photorhabdus luminescens *reference genome. **(b) **Schematic reconstruction of I-*Ceu*I pulsed field gel electrophoresis (PFGE) patterns for TT01_/I _and the six variants representing gels presented in Additional data file 2. Fragment sizes were calculated using the TT01_/I _genome as a reference. Lane 1: TT01_/I_. Lane 2: TT01_/II_. Lane 3: TT01α_/I_. Lane 4: TT01α_/II_. Lane 5: TT01α'_/II_. Lane 6: VAR*. Lane 7: REV. **(c) **Clustering of the PFGE patterns. Patterns were compared using the Dice coefficient for each pair. Patterns were clustered by UPGMA.

We examined the whole genome architecture of each variant using I-*Ceu*I genomic macrorestriction and pulsed field gel electrophoresis (PFGE) in order to detect large rearrangement such as deletions and amplifications by recombination between *rrn *or deletions, amplifications, and translocations inside I-*Ceu*I fragments. I-*Ceu*I is an intron-encoded enzyme that specifically cleaves a 26-base-pair site in the bacterial 23S rRNA gene. The PFGE pattern obtained for the TT01_/I _strain matched the pattern of I-*Ceu*I fragments predicted from the complete TT01_/I _genome sequence (Figure 3a, b; also see Additional data file 2 for the details of the gels). Using the TT01_/I _pattern used as a reference, we observed large genomic rearrangements in TT01α_/I_, TT01α_/II_, TT01α'_II_, VAR*, and REV. PFGE patterns revealed identical profiles for primary and secondary PVs within both TT01 and TT01α lineages (Figure 3b and Additional data file 2). Therefore, PV status (primary versus secondary) in these variant lineages is independent from global genomic architecture.

Cluster analysis of the seven observed I-*Ceu*I patterns reveals that variant lineages are in fact genomic lineages (Figure 3c). The TT01 and TT01α lineages exhibit genomic homogeneity. The TT01α' lineage shared common genomic features with the TT01α lineage, but exhibited a more polymorphic genomic pattern than TT01 and TT01α lineages.

The PFGE patterns of TT01α and the TT01α' lineages only reveal six apparent I-*Ceu*I fragments, instead of seven fragments in the TT01_/I _reference chromosome; however, the intensity of the 295-kb band suggests that it may represent two different fragments. We used Southern blot analysis to confirm that the seven *rrn *copies are present in all the variants (Additional data file 3). Therefore, variation in I-*Ceu*I PFGE patterns among the TT01 variants appeared to be unrelated to deletion or amplifications mediated by recombination between *rrn *operons.

Additionally, the 465 kb faint band in the TT01α'_/II _pattern (white star in Additional data file 2) corresponded to a fragment in the REV pattern, suggesting the existence of a 'REV-like' chromosome subpopulation in TT01α'_/II_.

### Deletions and amplifications in the TT01α_/I _and VAR* variants, representative of the TT01α and TT01α' lineages

Large genomic rearrangements were present in the TT01α and TT01α' lineages. We further evaluated the nature of these rearrangements by comparing gene content between representative variants of each lineage, TT01_/I_, TT01α_/I _and VAR*, using genomic DNA hybridization on a *P. luminescens *TT01_/I _microarray.

**Figure 4:**
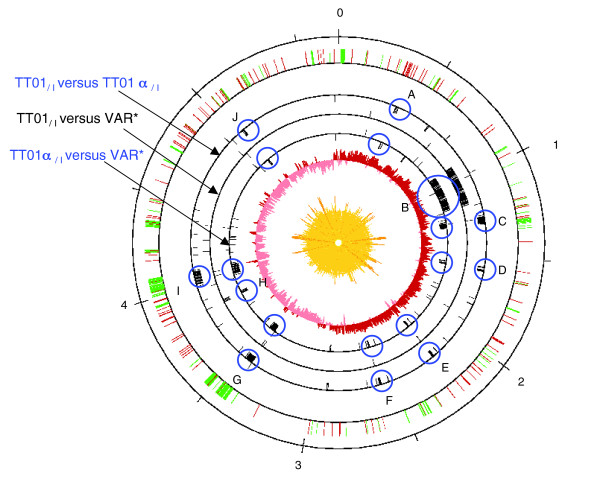
Schematic representation of DNA microarray data as a circular map of the TT01_/I _genome. Circle 1 (from outside to inside): scale marked in megabases. Circle 2: location of transposases (red) and phage-related genes (green) location. Circles 3, 4, and 5: DNA microarray data comparing TT01_/I _and TT01α_/I _genomes (circle 3), TT01_/I _and VAR* genomes (circle 5), and synthesis from both experiments (circle 4). Deleted genes are represented by bars inside the circle. Amplified genes are represented by bars outside the circle. Deleted and amplified regions are circled in blue. Circle 6: GC bias (G-C/G+C). Circle 7: GC content with <32% G+C in light yellow, between 32% and 53.6% G+C in yellow, and with >53.6% G+C in dark yellow.

Totals of 159 and 162 genes were absent from TT01α_/I _and VAR*, respectively (see Additional data file 4). We located these genes on a circular map of the TT01_/I _chromosome (Figure 4); they mostly clustered into eight regions absent from both the TT01α_/I _and VAR* genomes (regions A, C, D, E, F, G, I, and J) and one region specifically absent from the VAR* genome (region H). The deleted regions were located throughout the chromosome, with no particular symmetry around the replication origin or termination site. Several regions displayed a GC bias inversion (C, D, E, G, I, and J). Three overlapped with phagic regions (C, G, and I), suggesting that prophage excision occurred during the TT01_/I _to TT01α_/I _transition (Table 2). As well as phagic genes, the deleted regions encompass putative mobile and recombination-mediating elements such as insertion sequences and recombination hotspot (Rhs) elements (region A), and plasmid-related protein-encoding genes (region J)(Table 2). The regions C, D, E, and F potentially encode peptide synthetases involved in antimicrobial compound synthesis (Table 2). However, we did not observe any significant difference in antimicrobial activity between TT01_/I _and TT01α_/I _tested for 14 indicator strains (data not shown).

A more thorough analysis of hybridization ratios revealed that 122 genes had a ratio higher than 1.4 in the VAR* genome (Additional data file 5). In contrast, comparison of the TT01_/I _and TT01α_/I _genomes revealed only four genes with a ratio higher than 1.4. These findings suggest that numerous genes are amplified in the VAR* genome. Among these potentially amplified genes, 112 are clustered in a unique and large 275-kb region, named B. This region encompasses 4.8% of the TT01_/I _genome (from plu0769 = *mrfA *to plu0980 = *hpaA*; Figure 4). Region B is located within the first quarter of the TT01_/I _chromosome and is not delimited by obvious repeat elements. According to TT01_/I _genome annotations, the region B may be involved in numerous and different functions (Table 2): basal cellular functions involving the DNA polymerase III ε chain (plu0943 = *dnaQ*), enolase (plu0913 = *eno*), and proteins involved in tryptophan metabolism (plu0799 = *tnaA*; plu0800 = *mtr*); and environment and/or host interactions, involving the major fimbrial biosynthesis locus (plu0769-0778 = the *mrfABCDEFGHJ *operon), insecticidal toxin proteins (plu0805 = *tccA3*; plu0806 = *tccB3*; plu0960 = *tcc2*; plu0961 = *tcdB1*; plu0962 = *tcdA1*; plu0964 = *tccC5*; plu0965 = *tcdA4*; plu0970 = *tcdB2*; plu0971 = *tcdA2*), and proteins similar to pyocins (plu0884; plu0886-0888; plu0892; plu0894).

**Table 2:** Deleted and amplified regions in the TY01α_/I _and VAR* genomes

Locus	Probable nature of event	Gene region	Size (in kb)	Products of interest (similarity or function)	Matching GI^a ^or EVR^b^
A	Deletion	plu0338-plu0355	18	DNA cytosine, ethyl-transferase, mismatch repair endonuclease, unknown proteins, Rhs proteins, IS630 family	Part of GI plu0310-plu0373
B	Amplification	plu0769-plu0980	275	Proteins involved in basal metabolism (DNA polymerase III ε chain, enolase, tryptophan metabolism) and in interaction with environment and/or host (fimbrial biosynthesis, Tc insecticidal toxins, pyocins)	Encompassed GI plu0884-plu0901, GI plu0914-plu0938, and overlapped a part of GI plu0958-plu1166
C	Deletion	plu1086-plu1123	44	Unknown proteins, phage regulators, peptide synthetase, transposase, bacteriophage proteins	Part of GI plu0958-plu1166
D	Deletion	plu1861-plu1876	12	Antibiotic biosynthesis	Part of GI plu1859-plu1894
E	Deletion	plu2191-plu2200	11	Antibiotic synthesis and transport	Part of EVR plu2179-plu2224
F	Deletion	plu2468-plu2476	8	unknown protein, ABC transporter, toxoflavin biosynthesis, transposase	EVR plu2468-plu2476
G	Deletion	plu2874-plu2960	54	Bacteriophage proteins	Part of GI plu2873-plu3038
H	Deletion	plu3238-plu3252	22	Unknown proteins, VgrG proteins	Part of GI plu3207-plu3275
I	Deletion	plu3380-plu3504	89	Bacteriophage proteins	Part of GI plu3379-plu3538
J	Deletion	plu4324-plu4328	12	Unknown and plasmid-related proteins	EVR plu4319-plu4332

^a ^Genomic islands described in [42]. ^b ^Enterobacterial variable regions described in [56].

To determine whether DNA microarray experiments explain the architectural modifications observed by macrorestriction experiments, we compared the two sets of data. The observed I-*Ceu*I macrorestriction fragments from the TT01α lineage (36 kb, 295 kb, 295 kb, 330 kb, 465 kb, 610 kb, ~3600 kb) were similar to the theoretical I-*Ceu*I fragments calculated after size subtraction of the eight deleted regions from the TT01_/I _I-*Ceu*I fragments (36 kb, 244 kb, 266 kb, 330 kb, 462 kb, 627 kb, ~3478 kb). Therefore, large-scale deletion events appear to underlie the TT01 to TT01α lineage transition. DNA microarray experiments in the TT01α' lineage identified a 275 kb amplification of the TT01_/I _genome. Duplication or triplication of region B may account for the increase in genome size (~100 kb to 650 kb) observed by macrorestriction for the TT01α to TT01α' transition. Therefore, duplication appears to be mainly responsible for the TT01α to TT01α' lineage transition.

### Homologous recombination between long repeats led to serial deletions of the region H in the TT01α and TT01α' lineages

**Figure 5:**
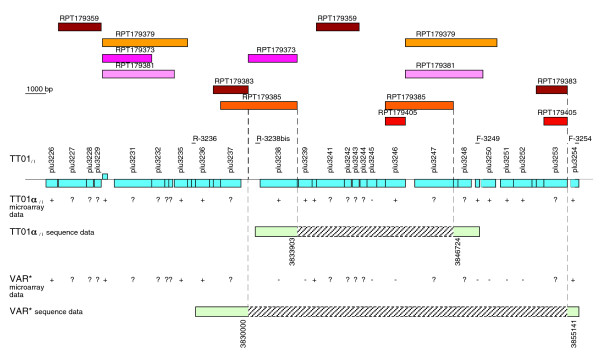
Successive deletions between homologous repeats in the region H. Genetic map of TT01_/I _region H is shown (blue boxes are open reading frames [ORFs]). Location of repetition units (RPT) larger than 1 kilobase (kb) is indicated (hatched colored boxes). RPT were systematically searched on the whole TT01_/I _genome sequence by using Nosferatu, software that can detect approximate repeat sequences [46]. The RPTs are numbered according their position on the chromosome. DNA microarray data for the TT01α_/I _and VAR* genomes are indicated. '+': the gene is present. '-': the gene is absent. '?': the gene is not represented on the microarray. Schematic representation of TT01α_/I _and the VAR* variant deletions is shown. Deletion borders were obtained from sequencing between the R-3236 and F-3254 primers in the VAR* variant, and between the R-3238bis and F-3249 primers in the TT01α_/I _variant. Green and hatched gray boxes represent regions in TT01α_/I _and VAR* genomes variants that were found to be present or absent, respectively. The deleted regions encompassed sequence between coordinates 3.833.904 and 3,846,724 in TT01α_/I _genome and coordinates 3,830,001 and 3,855,141 in VAR* genome.

We first examined the genomic deletions observed in the TT01α_/I _and VAR* variants. We focused on region H, which, by contrast to other deleted regions, did not exhibit typical recombination-mediating elements. Probes targeting different parts of the region H were hybridized on genomic DNA of the wild-type strain and the six variants. Hybridization patterns were identical within each variant lineage and confirmed the presence of a 25 kb deletion within the region H (from *plu3237 *to *plu3253*) for the TT01α' lineage (data not shown). Southern analysis also indicated the presence of a small deletion of about 10 kb (from *plu3238 *to *plu3248*) in the TT01α lineage. To map the deletion borders accurately, primers flanking the 25-kb deletion (R-3236 and F-3254) and the 10-kb deletion (R-3238bis and F-3249) were designed (Figure 5) and used for PCR amplification in the TT01α' and TT01α lineages. Amplified fragments of 4.8 kb and 5.2 kb were observed (data not shown). These fragments were sequenced for TT01α_/I _and VAR*, and the deletion was physically mapped (a genetic map of the region H is presented in Figure 5). The deletions in TT01α_/I _and VAR* were 12,820 bases (from coordinates 3,833,904 to 3,846,723) and 25,140 bases long (from coordinates 3,830,001 to 3,855,140), respectively.

We used Nosferatu, software that can detect approximate repeats in large DNA sequences [46]. The region H is rich in pairs of repetition units (RPT) larger than 1 kb (Figure 5). Each deletion began at the right-hand extremity of the first repetition and finished at the right-hand extremity of the corresponding second repetition (RPT179385 repetitions for the 10-kb deletion and RPT179383 repetitions for the 25-kb deletion). Therefore, successive deletions mediated by homologous recombination between RPT are likely to have occurred in the region H during the TT01_/I _to TT01α_/I _to VAR* transition, leading to genomic reduction.

### A single block duplication of region B is specific to the TT01α' lineage

**Figure 6:**
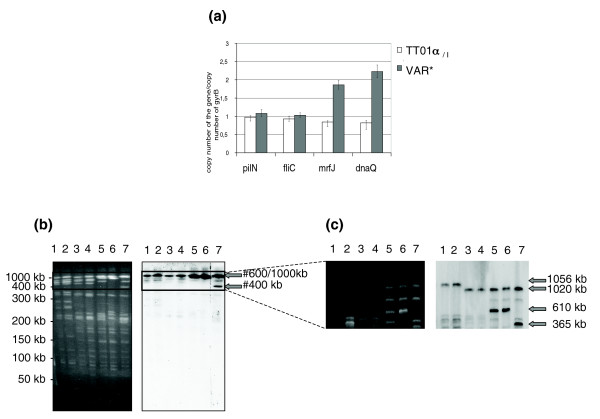
Duplication of region B. **(a) **Quantitative PCR was carried out for *mrfA *(plu0769) and *dnaQ *(plu0943) using genomic DNA from TT01α_/I _and VAR* variants and specific internal primers for each gene. *pilN *(plu1051) and *fliC *(plu1954) were used for negative controls. PCR was performed in triplicate and data are presented as ratios, with *gyrB *as the control gene (95% confidence limits). **(b, c) **Pulsed field gel electrophoresis (PFGE) of *Not*I-hydrolyzed genomic DNA from TT01α_/I _and the six variants following by Southern blot and hybridization with a probe covering the region B (probe B). The PFGE conditions allow separation of *Not*I fragments between 50 and 400 kb (panel b) or between 350 and 1,350 kb (panel c). Gray arrows indicate fragments that hybridize with the probe B. Lane 1: TT01_/I_. Lane 2: TT01_/II_. Lane 3: TT01α_/I_. Lane 4: TT01α_/II_. Lane 5: TT01α'_/II_. Lane 6: VAR*. Lane 7: REV.

In a second set of analyses, we focused on the gene amplification observed in region B, occurring in the TT01α_/I _to VAR transition. Quantitative PCR was performed for two genes in region B, *mrfA *(plu0769) and *dnaQ *(plu0943). Comparison of VAR and TT01α_/I _data confirmed that these two genes were duplicated in the VAR* genome (Figure 6a).

In order to determine whether region B is duplicated specifically in the VAR* variant or in all variants of the TT01α' lineage, a probe covering the entire region B (the probe B) was prepared and hybridized to genomic DNA of the wild-type strain and the six variants. According to the TT01_/I _genome sequence, *Not*I hydrolysis generates 25 fragments with a unique 1,056-kb fragment containing region B. Hybridization of the probe B to *Not*I-hydrolyzed genomic DNA generated a unique fragment of 1,056 kb in the TT01 lineage and of 1,020 kb in the TT01α lineages (Figure 6b, c). By contrast, in the TT01α' lineage, the B probe hybridized to the 1,020-kb fragment and an additional fragment. This second fragment has a similar size in TT01α'_/II _and VAR* variants (610 kb) but is smaller (365 kb) in the REV variant. These findings showed that duplication of region B occurred in all TT01α' lineage variants.

Region B encompasses 275 kb in the TT01_/I _genome sequence; thus, we determined whether the resulting amplified genes were dispersed in the genome or co-localized in an unique block. The unique additional fragment detected by the probe B in the TT01α'_/II _and VAR* variants indicated that the product of the region B amplification is constituted either of one block or a few blocks co-localized in a genomic region whose size is smaller than 610 kb in TT01α'_/II _and VAR* and smaller than 365 kb in REV. The probe B was also hybridized to *Apa*I-hydrolyzed genomic DNA of the wild-type strain and the six variants. The seven patterns were identical and the probe B hybridized with the two main 74 and 156 kb fragments covering the major part of region B according to the TT01_/I _genome reference sequence (data not shown). Because the duplication did not modify the *Apa*I restriction pattern, we concluded that region B was amplified as a single block.

## Discussion

### Variant lineages are genomic lineages characterized by extensive genomic rearrangements

Our study provides the first extensive investigation into genomic rearrangements in *Photorhabdus *variants. First, we evaluated phenotypic traits of the three variant lineages (Figure 1). The TT01 lineage is derived from the TT01_/I _strain, which was isolated from the *H. bacteriophora *TH01 nematode collected in Trinidad in 1993 [43] and whose genome is sequenced [42]. The TT01α lineage is derived from the TT01α_/I _genomic variant, which was collected from *H. bacteriophora *TH01 maintained and multiplied in the laboratory. The TT01α' lineage was derived from the TT01α_/I _variant after prolonged culture in synthetic medium. Each lineage is composed of PVs, whereby the primary form is characterized by the presence of typical phenotypic traits that are absent from the secondary form. The TT01α' lineage has an additional level of complexity, because the PVs exhibit features of CVs such as unstable morphotypes.

We then examined the genomic architecture of each variant in macrorestriction experiments and used comparative DNA microarray hybridization experiments to analyze the genomic content of representative variants for each lineage. Our findings revealed that large genomic rearrangements characterize each variant lineage. Consequently, these findings provide insight into probable scenarios underlying each lineage transition. The whole-genome organization of the TT01 lineage is described by the TT01 reference genome [42]. Large-scale deletion events in the TT01 flexible gene pool seem to be involved in the TT01 to TT01α lineage transition. Deletion events in the TT01 flexible gene pool and a single block duplication encompassing 4.8% of the TT01 reference genome appear to underlie the TT01 to TT01α' lineage transition. The genomic clusters do not depend on the PV status (see below). Thus, each variant lineage is a genetic lineage.

### Deletion at new recombination hotspot

To explain the molecular mechanisms involved in the rearrangements in our variants, we investigated potential repetitive elements and recombination-mediating elements flanking the rearranged regions. Large genomic architectural changes are often driven by homologous recombination between repeated sequences. The nature of the change then depends on the relative orientation, size, and spacing of the repeated sequences [47-50]. Recombination events often occur at the *rrn *operon in Gram-negative bacteria, such as *Salmonella*, *Rhizobium*, *Escherichia coli*, and *Ochrobactrum *[11,13,18,51,52]. However, despite the variation detected in PFGE analysis of the *rrn *skeleton for the three variant lineages, we demonstrated that the rearrangements are not the result of *rrn *recombination.

Apart from homologous recombination, rearrangements can be induced by site-specific recombination, associated with recombination-mediating elements such as mobile elements, or by illegitimate recombination, linked to shortly spaced repeats [49,50]. Most of the deleted regions in the TT01α lineage are rich in potential rearrangement-mediating elements, with both repeated sequences - including insertion sequences and Rhs elements - and mobile elements, including phagic and plasmid-related genes.

Genomic annotation of the region H, which underwent successive deletions in the TT01α_/I _and VAR* variants, did not describe the presence of typical repetitive or recombination-mediating elements. The region H belongs to a large genomic island containing the genes *vgr *and *hcp*, initially described as genes associated with *Rhs *elements. *Rhs *elements are repeated sequences in the *E. coli *genome that mediate major chromosomal rearrangements [51,53,54]. Although the TT01_/I _genome contains *Rhs*-like elements [42], no *Rhs *element is located in the genomic island encompassing the region H. Nevertheless, we identified pairs of approximate long repeated sequences (>1 kb) in direct orientation (RPT) that corresponded to the observed deletion junction points. Therefore, successive deletions in the region H are likely to have been mediated by homologous recombination between RPT during the transition from TT01_/I _to TT01α_/I _to VAR*, leading to genomic reduction.

There was a strong selective pressure during the TT01α to TT01α' lineage transition (3 months in LB broth without shaking). This environmental constraint could thus be responsible for the rearrangement leading to the region H deletion. However, the region H deletion was already initiated during the former transition (TT01_/I _to TT01α_/I _in the laboratory-maintained nematode). Therefore, the observed reduction genomic size is more likely to be the result of particular genomic features (the RPT) rather than environmental constraints.

The region H is unique in the TT01_/I _genome. Nevertheless, some RPT elements have similarities with sequences elsewhere in the TT01_/I _genome, in the *Photorhabdus *strain W14 genome [55] or in other Enterobacteriaceae genomes such as *Yersinia pseudotuberculosis *IP32953 (BX936398.1), *Yersinia pestis *Angola (CP000901.1), *Yersinia pestis *Pestoides F (CP000668.1), *Yersinia pestis *CO92 (AL590842.1), *Yersinia pestis *biovar Microtus str. 91001, (AE017042.1, *Yersinia pestis *Antiqua (CP000308.1), *Yersinia pestis *Nepal 516 (CP000305.1), *Yersinia pestis *KIM (AE009952.1), *Yersinia pseudotuberculosis *IP 31758 (CP000720.1), and *E. coli *CFT073 (AE014075.1). Therefore, we propose that the region H represents a new type of bacterial recombination hot spot, which is *vgr*- and *hcp*-rich, but lacks *Rhs *elements.

### A new duplication class

We described a single block duplication (region B) targeting a 275-kb region of the TT01_/I _genome in the TT01α' lineage. This significant duplication encompasses 4.8% of the TT01_/I _genome. Region B is not located near the replication origin or termination and does not correspond to genomic islands or enterobacterial variable regions previously identified [42,56]. GC content or GC skew deviations are not evident.

Gene amplifications can occur through three kinds of known mechanism: homologous recombination between direct repeats, illegitimate recombination, or escape replication. No repeated elements flanking region B were detected, despite the use of the Nosferatu software [46], excluding the possibility of homologous recombination underlying this duplication. Region B duplications may result from illegitimate recombination between short repeats [47,57,58]. However, amplification copy number resulting from illegitimate recombination events is often high, even for large amplicons, such as in *Acinetobacter *sp. ADP1 or *Streptomyces kanamyceticus *[59,60]. Escape replication involves amplification of large regions of the host genome (several hundred kilobases), next to phage integration sites after induction of the phage lytic cycle [61-64], or around degraded prophages without the induction of specific phage lysis [65]. Although phage remnants represent 4% of the *Photorhabdus *genome [42], lytic phages have not been identified in *Photorhabdus *strains, even after extensive investigation of lytic induction conditions [66]. We detected the presence of an 11-kb phagic segment (plu0818-plu0826) in region B, potentially representing a degraded prophage. However, whereas the copy number usually resulting from the escape replication mechanism ranges between three and ten, with its intensity decreasing symmetrically from the center, region B in the TT01α' lineage genomes represents a single block homogeneous duplication. We only identified one other previously reported example of a large duplication without repeated flanking sequences - a 250 kb duplication in *Mycobacterium smegmatis *mc^2 ^155 genome [67]. Therefore, the duplication of region B is likely to belong to a new class of duplications.

### Observed phenotypes and global genomic architecture are not systematically correlated

Large genomic changes such as deletions and duplications are supposed to have important fitness effects. In our study, we firstly demonstrated that the PV status (primary or secondary) is independent from global genomic architecture. This was consistent with previous studies analyzing specific genetic regions [28,38,39] and with partial genome studies [33,40,41], but this is the first time it has been demonstrated using a whole-genome approach.

We showed that the overall genomic pattern corresponds to the variant lineage. Both the phenotype and pathogenic traits of the primary PV (or the secondary PVs) are indistinguishable between the TT01 and TT01α lineages. Therefore, changes in the genomic architecture of these strains did not lead to observable changes in the phenotype. Furthermore, certain regions that were deleted in the TT01α lineage potentially encode biosynthesis pathways for antimicrobial compounds. However, we did not observe any difference in antimicrobial activity between TT01_/I _and TT01α_/I_. This finding suggests that some TT01_/I _genes are redundant. Indeed, genes encoding proteins potentially involved in the biosynthesis of antimicrobial compounds are over-represented in TT01_/I _genome [42]. Moreover, the encoded proteins in the deleted regions may be adaptive factors required for specific conditions that are not encountered in the laboratory or in our antibiosis assays.

The TT01α' lineage differs from the two other lineages due to its polymorphic genomic pattern. Furthermore, this lineage is composed of three unstable CVs and the virulence of the stabilized VAR* variant is attenuated in insects. This is consistent with previously reports of CVs isolated from the *Photorhabdus *genus [33]. Therefore, changes in genomic architecture might be correlated to phenotypic changes in variants of this lineage. The main rearrangement observed in the TT01α' lineage is the region B duplication. According to TT01_/I _genome annotation, region B may be involved in both basal cellular functions and environment and/or host interactions. Gene duplication events can underlie modification of phenotypes [58]. However, we did not detect any modification of gene transcription in region B using transcriptomic microarray comparison between the VAR* and TT01α_/I _variants (Gaudriault S, unpublished data). Thus, this duplication does not appear to modify gene expression in the VAR* variant. Therefore, the attenuation of virulence of the VAR* variant is not likely to be due to amplified expression in region B. Rather, it is more likely that the 'cost' to the bacteria of the increased genome size is decreased virulence in insects.

We conclude that the observed phenotypes and overall genomic architecture are not systematically correlated in TT01, TT01α, and TT01α' lineages. It is likely that this result is general in the field of bacterial genomic architecture. Similar observations were previously made between strains of the *Pseudomonas aeruginosa *species [68], but also inside a clonal bacterial population of a wide range of bacterial groups such as *Yersinia pestis *[19], *Pseudomonas aeruginosa *[17], and *Sinorhizobium meliloti *[16].

### Stability and plasticity of bacterial genome architecture

Do large genomic rearrangements occur randomly or are they shaped by drastic selective evolutionary forces? Several years of comparative genomics between whole bacterial genomes showed that the prokaryotic genome is a heterogeneous entity, with regions of stability and flexibility [4,49,50]. Genomic stability is subject to selective pressures such as functional replication [69], gene essentiality [70], or translation [71]. The three main routes of evolution of genome repertoire are lateral gene transfer, when several bacterial communities share a same ecological niche, deletions, and duplications [4,49,50]. The dynamism of genome repertoire inside a clonal population only arises by the last two phenomena, as illustrated by our study on *Photorhabdus *variants.

In *E. coli*, the chromosome is organized in structured macrodomains, limiting genome plasticity. Whereas some genomic rearrangements between these macrodomains have only moderate effects on cell physiology, others have detrimental effects [72]. The rearrangements that we observed in our variants may have been selected to preserve chromosomal configurations that are not detrimental for bacterial fitness in the laboratory or in the nematode. We believe that structured macrodomains that restrict chromosome plasticity are likely to exist in other bacterial genus. Identification of structured macrodomains in *P. luminescens *genome would provide better knowledge on evolutionary forces modeling bacterial genome.

### Clonal variation, environmental adaptation, and bacterial evolution

The major genomic variations described in TT01 variants have cryptic consequences in our laboratory conditions. The absence of associated phenotypes makes them difficult to identify, explaining why such genomic variations are rarely observed. However, further studies of such genomic variations may be crucial for a better understanding of bacterial adaptation and evolution.

Indeed, we observed that the extensive genomic rearrangements in *Photorhabdus *variants were often associated with several genomic subpopulations in the same culture. Similar observations were previously made for a *P. luminescens *TT01_/I _locus encoding a phage tail-like structure [73] and the *mrf *locus of the *P. temperata *strain K122 [74]. In *Sinorhizobium meliloti*, *Yersinia pestis*, and *Pseudomonas aeruginosa*, extensive variations of genome architecture, without obvious changes in phenotype, were also observed during bacterial growth in broth medium [16,17,19]. Different pre-existing chromosomal forms in a clonal bacterial population are likely to give this population an adaptive capacity. It is therefore possible that bacterial populations maintain various subpopulations with different genomic structures as a way to cope with different environments during its life cycle.

Additionally, deletion events in TT01α and TT01α' lineages are located within the TT01_/I _'flexible' gene pool. Whereas intragenomic recombination in the 'flexible' gene pool have been widely studied using comparative genomics for different bacterial genera, species, and strains [1-4], similar reports for clonal variants are rare. Gene repertoires of the 'flexible' gene pool may evolve through variations in bacterial subpopulations and then become fixed after bacterial speciation. Such pre-existing or currently existing genomic variations have an important role in evolutionary patterns of natural eukaryotic populations [75]. They may also have a determinant role in bacterial evolution.

## Conclusion

The study of molecular mechanisms underlying genomic plasticity in clonal populations is challenging because classical molecular tools only detect the major genomic state of the population. Such studies are easier in bacterial species with a high rate of bacterial variants. With our model, *P. luminescens*, we identified two new genomic rearrangements, allowing a new research axis for gaining a comprehensive knowledge of bacterial chromosome plasticity. The cryptic consequences of large genomic rearrangements in our model also allow prospective comprehensive analysis of bacterial genome evolution. Therefore, we propose that the *P. luminescens *TT01 strain represents a new bacterial model for study of genomic plasticity.

## Materials and methods

### Strains, plasmids, primers, and culture media

All bacterial strains and plasmids used in this study are listed in Additional data file 6. Primers are listed in Additional data file 7. *P. luminescens *was grown at 28°C in LB broth or on nutrient agar 1.5% (BD Difco™, Franklin Lakes, New Jersey, USA) for 48 hours. *Escherichia coli *was grown at 37°C in LB broth or on LB supplemented with 1.5% agar (BD Difco™, Franklin Lakes, New Jersey, USA). Strains were stored at -80°C in LB broth containing 16% glycerol (vol/vol). Secondary variants were obtained by prolonged culture of primary variants at 28°C for 10 days in Schneider's insect medium (Cambrex Bio Science, Walkersville, Maryland, USA) with shaking (TT01_/II _[30]), for 10 days in LB broth with shaking (TT01α_/II_), or for 3 months in LB broth without shaking (TT01α'_/II_). Secondary variant phenotypes were evaluated from culture on NBTA (nutrient agar 1,5%, 25 mg/l bromothymol blue and 40 mg/l triphenyl-2,3,5-tetrazolium chloride) plates and on TreGNO plates (see below) at 28°C. Secondary variants were identified by performing phenotypic tests as previously described [76] and controlled by PCR-restriction fragment length polymorphism of the 16S rRNA gene [77].

### Analysis of phenotypic variants on a new selective medium: TreGNO

*Xenorhabdus *and *Photorhabdus *secondary variants are typically selected on NBTA plates to distinguish red secondary variants colonies from blue primary colonies [76]. Because of the high level of pigmentation of *Photorhabdus *colonies, the use of color assays does not allow clear distinction between primary and secondary variants for *Photorhabdus *genus. We found that TT01 secondary variants were able to undergo trehalose fermentation, whereas primary variants can not. On nutrient agar plates supplemented with trehalose (10 g/l) and bromothymol blue (25 mg/l), secondary colonies acidified the bromothymol blue and became yellow at 28°C after 48 hours. Primary colonies remained green. Furthermore, secondary colonies were flat and large with irregular borders. This new medium was named TreGNO medium and was routinely used for the discrimination of *Photorhabdus luminescens *strain TT01 phenotypic variants.

### PFGE and DNA electrophoresis

Intact genomic DNA was extracted in agarose plugs as follows. Bacterial cells grown on nutrient agar plates were suspended in phosphate-buffered saline (GIBCO^® ^Invitrogen, Carlsbad, California, USA) to a turbidity of 1.25 at 650 nm, included in 1% (vol/vol) low melting agarose (SeaPlaque^® ^GTG, FMC BioProducts, Rockland, Massachusetts, USA) solution and then subjected to lysis as described previously [78].

*Not*I and *Apa*I hydrolysis were performed by incubation of the agarose plugs overnight with 40 units of the endonuclease in buffer recommended by the supplier (New England Biolabs, Hertfordshire, UK), at 37°C for *Not*I and 25°C for *Apa*I. PFGE was carried out in a contour-clamped homogeneous field electrophoresis apparatus CHEF-DRII (Bio-Rad, Hercule, California, USA) in a 0.8% agarose gel in 0.5× Tris-borate-EDTA (TBE) at 10°C. PFGE conditions were as follows: for *Not*I fragments, a 35 to 5 second pulse ramp for 47 hours followed by a constant pulse time of 50 seconds for 6 hours at 4.5 V/cm; and for *Apa*I fragments, 35 to 5 seconds for 35 hours, followed by 5 seconds to 1 second for 4 hours at 4.5 V/cm.

I-*Ceu*I hydrolysis was performed as described previously [79]. For the separation of I-*Ceu*I fragments, different electrophoresis conditions were selected according to fragment size: a pulse ramp from 5 to 50 seconds for 24 hours at 6 V/cm for fragments with size below 700 kb; and a pulse ramp from 150 to 400 seconds for 45 hours at 4.5 V/cm for I-*Ceu*I fragments for fragments between 700 kb and 1 megabase. For I-*Ceu*I fragments larger than 1 megabase, PFGE was performed on Rotaphor apparatus (Biometra, Goettingen, Germany) using 0.7% agarose gels in 0.5× TBE buffer. The electrophoresis conditions used were as follows: 50 to 47 V (linear ramp), 6,000 to 1,000 seconds decreasing pulses (logarithmic ramp), with a increasing angle from 96 to 105°, buffer temperature 11°C, for 240 hours. I-*Ceu*I PFGE patterns were compared by calculating the Dice coefficient for each pair [80]. Patterns were clustered by UPGMA using the Phylip program package [81].

*Hin*dIII-hydrolyzed DNA was subjected to electrophoresis for 3 hours at 2.6 V/cm in a 0.8% agarose gel in 0.5× TBE using SubCell apparatus (Bio-Rad) [13].

### Southern blotting, probes, and hybridization experiments

Electrophoresis gels were transferred onto a Nytran N SuperCharge nylon membrane (Schleicher and Schuell, Dassel, Germany) by vacuum blotting in 20 × SSC (Euromedex, Souffelweyersheim, France).

A digoxigenin-labeled probe targeting 16S rRNA gene was obtained by PCR from genomic DNA of *P. luminescens *strain TT01_/I_, using primers 27f and 1492r with a dNTP mixture containing 0.1 mmol/l digoxigenin-dUTP [13].

Probes B and H were obtained using respectively small fragment insert from plg2711 and large fragment inserts from plbac4g08, plbac6h12, plbac3a10, plbac3c04, and plbac2f12. Fragment inserts were purified, sonicated into fragments of between 1 and 10 kb if insert size was higher than 10 kb, and labeled with digoxygenin by random priming (Dig DNA labeling Kit; Roche, Meylan, France). Hybridization of the probes was detected using a CSPD chemiluminescent system (Roche).

### Standard DNA manipulations

Genomic DNA was extracted as previously described [56] and stored at 4°C. We PCR-amplified the *lop*T1 deletion region with Taq polymerase (Invitrogen, Carlsbad, California, USA), in accordance with the manufacturer's recommendations, using the P*lopT1*.fw an P*lopT1*.rev primers. The region H was amplified by PCR with the Herculase Enhanced DNA polymerase (Stratagene, Amsterdam Zuidoost, Pays Bas), in accordance with the manufacturer's recommendations, using the R-3236, F-3249, R-3238bis, and F-3254 primers. For sequencing region H deletions, we purified the 4.8 kb and 5.2 kb fragments using the Montage PCR kit (Millipore, Guyancourt, France) and sequenced using PCR primers and chromosome walking (Millegen, Toulouse, France). Sequencing of the 5.2 kb fragment central region of the fragment failed probably because of the presence of repetitions. A 3.2 kb central region was therefore amplified by PCR with *Pst*IdMutF and *Xba*IdMutR primers. The amplicon was hydrolyzed by *Pst*I and *Xba*I, ligated into *Pst*I- and *Xba*I-hydrolyzed pUC19, and inserted into *E. coli *XL1blue by transformation. The resulting plasmid was purified by Nucleobond AX-100 kit (Macherey-Nagel, Hoerd, France), and the insert was sequenced with *Pst*IdMutF and *Xba*IdMutR primers and then by chromosome walking.

### DNA microarray hybridization and analysis

DNA microarray hybridization and analysis were performed as previously described [56].

### Quantitative PCR analysis

Quantitative PCR was performed in triplicate using the LightCycler FastStart DNA Master^PLUS ^SYBR Green I kit from Roche Diagnostics with 1 ng genomic DNA and 1 μmol/l specific primers targeting *fliC *(L-1954 and R-1954), *mrfJ *(L-0778 and R-0778), *dnaQ *(L-0943 and R-0943), and *pilN *(L-1051 and R-1051). The enzyme was activated for 10 minutes at 95°C. Reactions were performed in triplicate at 95°C for 5 seconds, 60°C for 5 seconds and 72°C for 10 seconds (45 cycles), and monitored in the Light Cycler (Roche). Melting curves were analyzed for each reaction; all reactions exhibited a single peak. The amount of PCR product was calculated with standard curves obtained from PCR with serially diluted TT01_/I _genomic DNA. All data are presented as ratios, with *gyrB *(primers L-0004 and R-0004) as a control (95% confidence limits).

### Sequence analysis

Sequence annotation of the TT01_/I _genome was obtained from the MaGe database [82]. We evaluated amino-acid and nucleotide similarity using BLASTP and BLASTN software [83]. We used Repseek software, previously Nosferatu [46], to detect approximate repeats in large DNA sequences.

### Pathogenicity assays

*In vivo *infection assays were performed as previously described [45]. We performed three independent experiments for each variant. Statistical analysis were performed as previously described [84].

### Antibiosis plate assays

Antibiosis assays were performed as previously described [76] with the following bacterial species: *Micrococcus luteus*, *Staphylococcus epidermidis *CIP 6821, *Staphylococcus aureus *CIP 7625, *Escherichia coli *CIP 7624, *Proteus vulgaris *CIP 5860, *Pseudomonas aeruginosa *CIP 76.110, *Corynebacterium xerosis*, *Ochrobactrum intermedium *LMG 3301^T^, *Ochrobactrum anthropi *ATCC 49188^T^, *Ochrobactrum *sp. FR49, *Erwinia amylovora *CFBP1430, *Pseudomonas *sp. BW11M, *Salmonella enterica *14028s, and *Yersinia enterocolitica *serotype 08.

## Abbreviations

CV, colonial variant; kb, kilobase; NBTA, nutrient agar supplemented with bromothymol blue and triphenyl-2,3,5-tetrazolium chloride; PCR, polymerase chain reaction; PFGE, pulsed field gel electrophoresis; PV, phenotypic variant; Rhs, recombination hotspot; RPT, repetition units; SCV, small-colony variant; TBE, Tris-borate-EDTA; TreGNO, nutrient agar with trehalose and bromothymol blue.

## Authors' contributions

SG, SP, and AG characterized bacterial variants. SG, AL, and CL provided molecular materials. SG and AL performed microarray analysis. SG, CT, and EJ-B provided PFGE analysis. SG analyzed sequence data. SG wrote the paper with contributions from AG and EJ-B.

## Additional data files

The following additional data files are available with this paper. Additional data file 1 is a figure showing the deletion in the *lopT1 *gene in TT01_/I _strain and the six variants. Additional data file 2 is a figure showing PFGE of I-*Ceu*I-hydrolyzed genomic DNA of TT01_/I _strain and the six variants. Additional data file 3 is a figure showing the copy number of 16S rDNA in TT01_/I _and the six variants. Additional data file 4 is a table listing the TT01_/I _missing genes in TT01α_/I _and VAR* variants according whole-genome comparison using DNA microarray. Additional data file 5 is a table listing the TT01_/I _amplified genes in TT01α_/I _and VAR* variants, according to whole-genome comparison using DNA microarray. Additional data file 6 is a table listing strains and plasmids used in this study. Additional data file 7 is a table listing primers used in this study.

## Supplementary Material

Additional data file 1 Presented is a figure showing the deletion in the *lopT1 *gene in TT01_/I _strain and the six variants. Click here for file

Additional data file 2 Presented is a figure showing PFGE of I-*Ceu*I-hydrolyzed genomic DNA of TT01_/I _strain and the six variants. Click here for file

Additional data file 3 Presented is a figure showing the copy number of 16S rDNA in TT01_/I _and the six variants. Click here for file

Additional data file 4 Presented is a table listing the TT01_/I _missing genes in TT01α_/I _and VAR* variants according whole-genome comparison using DNA microarray. Click here for file

Additional data file 5 Presented is a table listing the TT01_/I _amplified genes in TT01α_/I _and VAR* variants, according to whole-genome comparison using DNA microarray. Click here for file

Additional data file 6 Presented is a table listing strains and plasmids used in this study. Click here for file

Additional data file 7 Presented is a table listing primers used in this study. Click here for file
