# Supplementary material for: Plastic architecture of bacterial genome revealed by comparative genomics of Photorhabdus variants
Source: Genome Biol. 2008 Jul 22;9(7):R117. doi: 10.1186/gb-2008-9-7-r117 (PMC2530875; doi:10.1186/gb-2008-9-7-r117)
Supplement: Additional data file 1 — Presented is a figure showing the deletion in the lopT1 gene in TT01/I strain and the six variants. [file gb-2008-9-7-r117-S1.pdf]

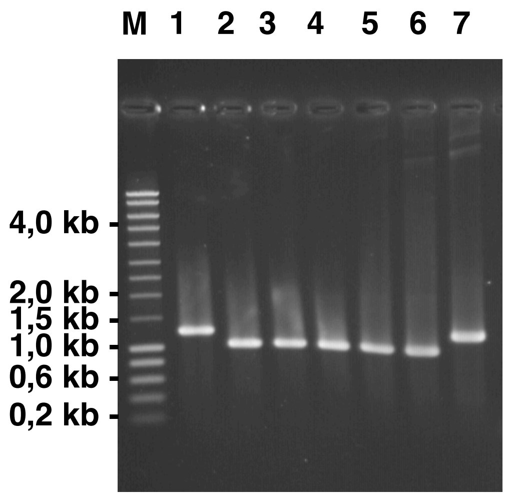

Additional data file 1 : Deletion in the *lopT1* gene in TT01$_{/I}$ strain and the six variants. PCR amplification were undertaken with the P*lopT1*.fw and P*lopT1*.rev primers and the resulting products were separated on TAEx1 agarose 1% gel. M : DNA ladder. Lane 1 : TT01 $_{/I}$. Lane 2 : TT01α $_{/I}$. Lane 3: TT01α' $_{/II}$. Lane 4 : VAR*. Lane 5 : REV. Lane 6 : TT01α $_{/II}$. Lane 7 : TT01$_{/II}$.
