# Supplementary material for: Plastic architecture of bacterial genome revealed by comparative genomics of Photorhabdus variants
Source: Genome Biol. 2008 Jul 22;9(7):R117. doi: 10.1186/gb-2008-9-7-r117 (PMC2530875; doi:10.1186/gb-2008-9-7-r117)
Supplement: Additional data file 2 — Presented is a figure showing PFGE of I-CeuI-hydrolyzed genomic DNA of TT01/I strain and the six variants. [file gb-2008-9-7-r117-S2.pdf]

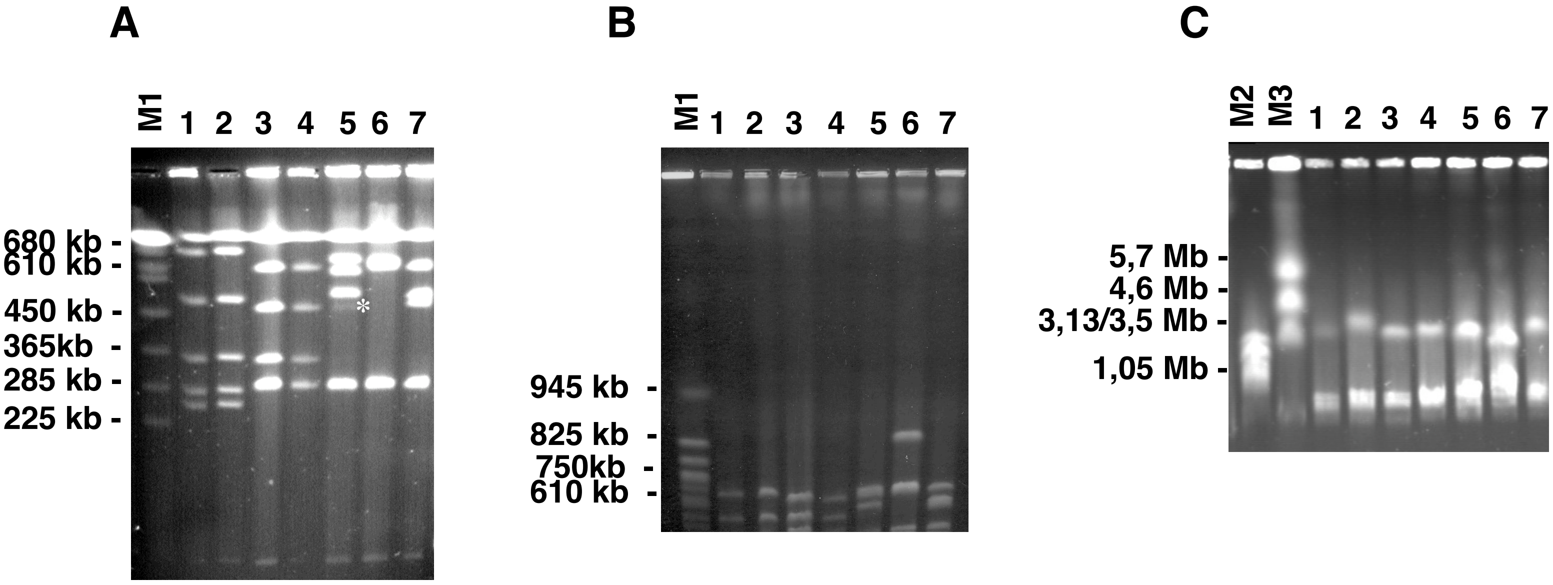

Additional data file 2 : Pulse field gel electrophoresis of I-*Ceu*I-hydrolyzed genomic DNA of TT01 wild-type strain and the six variants. Markers used are *Saccharomyces cerevisiae* chromosomes (M1), *Saccharomyces pombe* strain 972h- chromosomes (M2) and *Hansenula wingei* strain YB-4662-VIA chromosomes (M3). Lane 1 : TT01$_{/\mathrm{I}}$. Lane 2 : TT01$_{/\mathrm{II}}$. Lane 3: TT01α$_{/\mathrm{I}}$. Lane 4 : TT01α$_{/\mathrm{II}}$. Lane 5 : TT01α'$_{/\mathrm{II}}$. Lane 6 : VAR*. Lane 7 : REV.
