# Supplementary material for: Plastic architecture of bacterial genome revealed by comparative genomics of Photorhabdus variants
Source: Genome Biol. 2008 Jul 22;9(7):R117. doi: 10.1186/gb-2008-9-7-r117 (PMC2530875; doi:10.1186/gb-2008-9-7-r117)
Supplement: Additional data file 3 — Presented is a figure showing the copy number of 16S rDNA in TT01/I and the six variants. [file gb-2008-9-7-r117-S3.pdf]

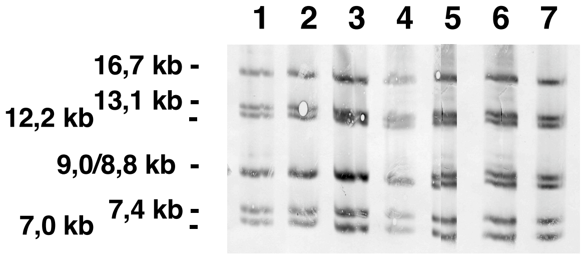

Additional data file 3 : Copy number of 16S rDNA in $TT01_{/I}$ and the six variants. Southern blot of *Hind*III-hydrolyzed genomic DNA were hybridized with a 16S rDNA probe. Sizes of hybridizing fragments were the $TT01_{/I}$ fragment sizes calculated using the genome sequence as reference. In the TT01 and TT01 α lineages, 5 unique bands and one double band were observed, and in the TT01 α' lineages seven bands were observed. This experiment confirmed that all the 7 *rrn* operons are conserved in all the variants as described for the reference genome $TT01_{/I}$. Lane 1 : $TT01_{/I}$. Lane 2 : $TT01_{/II.}$ Lane 3: $TT01\alpha_{/I}$. Lane 4 : $TT01\alpha_{/II}$. Lane 5 : $TT01\alpha'_{/II}$. Lane 6 : VAR*. Lane 7 : REV.
