# Supplementary material for: Plastic architecture of bacterial genome revealed by comparative genomics of Photorhabdus variants
Source: Genome Biol. 2008 Jul 22;9(7):R117. doi: 10.1186/gb-2008-9-7-r117 (PMC2530875; doi:10.1186/gb-2008-9-7-r117)
Supplement: Additional data file 4 — Presented is a table listing the TT01/I missing genes in TT01α/I and VAR* variants according whole-genome comparison using DNA microarray. [file gb-2008-9-7-r117-S4.pdf]

Additional data file 4. TT01/I missing genes in both TT01α/I and VAR* variants (green lines), in only TT01α/I variant (grey lines), in only VAR* variant (red lines) according whole-genome comparison using DNA micro-array.

Microarray position	plu	Name	Similarity	Protein	Ratio var/wt	Ratio mut/wt
11_95	EMBL_NAME=PLU0338	dcm		DNA-cytosine methyltransferase	0,348	0,362
12_285	EMBL_NAME=PLU0339	vsr		Very short patch repair protein (DNA mismatch endonuclease) (Vsr mismatch endonuclease) (V.EcoKDcn	0,456	0,486
2_65	EMBL_NAME=PLU0340		Some similarities with unknown protein		0,431	0,467
12_287	EMBL_NAME=PLU0342		Similar to unknown protein YobK of Bacillus subtilis		0,403	0,415
10_330	EMBL_NAME=PLU0348		Hypothetical gene		0,617	0,570
2_69	EMBL_NAME=PLU0355		Similar to VrgG protein		0,525	0,547
5_268	EMBL_NAME=PLU0535		Similar to unknown protein		0,298	0,319
12_81	EMBL_NAME=PLU0539		Some similarities with C-terminal region of hemagglutinin/hemolysin-related protein		0,572	0,580
1_263	EMBL_NAME=PLU0541		Some similarities with unknown protein		0,429	0,431
4_263	EMBL_NAME=PLU0863		Some similarities with unknown protein of Photorhabdus		0,337	0,390
11_359	EMBL_NAME=PLU0994	phfA	Some similarities with fimbrial adhesin precursor		0,332	0,356
9_376	EMBL_NAME=PLU1053	pilP	Some similarities with PilP protein		0,592	0,585
1_361	EMBL_NAME=PLU1075		Similar to unknown protein YhcG of Escherichia coli		0,272	0,306
12_341	EMBL_NAME=PLU1086		Similar to unknown protein		0,450	0,470
12_64	EMBL_NAME=PLU1088		Similar to unknown protein		0,396	0,432
12_339	EMBL_NAME=PLU1089		Some similarities with putative membrane protein		0,439	0,481
12_62	EMBL_NAME=PLU1090		Similar to putative membrane protein		0,412	0,436
10_351	EMBL_NAME=PLU1097		Some high similarities with unknown protein of Photorhabdus. Hypothetical protein with two candidate membrane-spanning segments		0,559	0,568
12_60	EMBL_NAME=PLU1105		Similar to aminotransferase		0,386	0,408
12_58	EMBL_NAME=PLU1106		Similar to L-mandelate and L-lactate dehydrogenase		0,404	0,436
10_347	EMBL_NAME=PLU1110		Some similarities with unknown protein		0,626	0,592
12_56	EMBL_NAME=PLU1111		Similar to reverse transcriptase/maturase		0,473	0,498
12_54	EMBL_NAME=PLU1113		Similar to peptide synthetase involved in antibiotic biosynthesis		0,499	0,515
12_52	EMBL_NAME=PLU1115		Similar to peptide synthetase involved in antibiotic biosynthesis		0,374	0,397
10_345	EMBL_NAME=PLU1117		Similar to C terminal part of peptide synthetase involved in antibiotic biosynthesis		0,641	0,532
10_62	EMBL_NAME=PLU1123		Some similarities with unknown protein of Photorhabdus. Hypothetical protein with two candidate membrane-spanning segments		0,463	0,491
12_194	EMBL_NAME=PLU1340		Highly similar to C-terminal region of RTX toxin RtxA		0,446	0,490
12_198	EMBL_NAME=PLU1342		Similar to N-terminal region of RTX toxin RtxA.		0,478	0,492
12_200	EMBL_NAME=PLU1343		Similar to C-terminal region of RTX toxin RtxA.		0,541	0,594
3_161	EMBL_NAME=PLU1483		Hypothetical gene		0,413	0,423
10_150	EMBL_NAME=PLU1514		Similar to tetracycline resistance protein TetA(P) homolog. Putative transmembrane protein		0,532	0,555
10_148	EMBL_NAME=PLU1515		Similar to MccB protein of Escherichia coli, involved in the production of microcin C7		0,533	0,561
12_340	EMBL_NAME=PLU1593		ATP-dependent Clp protease adaptor protein ClpS		0,381	1,119
12_202	EMBL_NAME=PLU1607		Hypothetical transmembrane protein		0,470	0,495
2_366	EMBL_NAME=PLU1751	ompN		Outer membrane protein N precursor (Porin OmpN)	0,449	0,494
11_162	EMBL_NAME=PLU1833		Some similarities with transmembrane domain protein		0,572	0,592
9_176	EMBL_NAME=PLU1834		Some weak similarities with unknown protein		0,539	0,572
7_164	EMBL_NAME=PLU1861		Truncated transposase		0,544	0,539
7_162	EMBL_NAME=PLU1865		Similar to phosphoenolpyruvate phosphomutase precursor (PEP mutase)		0,444	0,454
5_176	EMBL_NAME=PLU1866		Highly similar to nitrilotriacetate monooxygenase component A		0,531	0,589
5_174	EMBL_NAME=PLU1867		Similar to homocitrate synthase 2		0,633	0,565
9_291	EMBL_NAME=PLU1868		Similar to homocitrate sythase 1 and 2-isopropylmalate synthase (LeuA-1)		0,580	0,617
5_172	EMBL_NAME=PLU1869		Similar to large subunit of 3-isopropylmalate dehydratase, large subunit		0,366	0,406
2_278	EMBL_NAME=PLU1870		Similar to 3-isopropylmalate dehydratase, small subunit (leuD-2)		0,591	0,662
5_168	EMBL_NAME=PLU1873		Some similarities with NikS protein involved in antibiotic nikkomycin biosynthesis		0,375	0,412
5_166	EMBL_NAME=PLU1874		Some similarities with Nikkomycin biosynthesis protein, carboxylase		0,444	0,500
5_164	EMBL_NAME=PLU1875		Similar to integral membrane efflux protein. Putative secreted protein		0,422	0,458
5_162	EMBL_NAME=PLU1876		Hypothetical protein		0,346	0,382
2_375	EMBL_NAME=PLU2146		Similar to membrane protein YdbH of Escherichia coli		0,356	0,409
11_128	EMBL_NAME=PLU2191		Some similarities with diaminobutyric acid aminotransferase		0,605	0,590
2_98	EMBL_NAME=PLU2192		Some similarities with molybdopterin and thiamine biosynthesis protein		0,511	0,544
2_100	EMBL_NAME=PLU2193		Weakly similar to hypothetical gamma-butyrobetaine,2-oxoglutarate		0,426	0,433
2_102	EMBL_NAME=PLU2196		Some weak similarities with capsule biosynthesis protein		0,453	0,520
2_104	EMBL_NAME=PLU2197		Similar to NikS protein involved in the biosynthesis of the peptidyl nucleoside antibiotic nikkomycin		0,423	0,450
2_106	EMBL_NAME=PLU2198		Similar to the phosphoribosylamine--glycine ligase		0,559	0,634
2_108	EMBL_NAME=PLU2199		Similar to transporter		0,477	0,530
2_110	EMBL_NAME=PLU2200		Similar to unknown protein and to glutamyl-tRNA(Gln) amidotransferase chain A		0,547	0,580
6_98	EMBL_NAME=PLU2215		Similar to putative arginine methyltransferase		0,424	0,415
6_100	EMBL_NAME=PLU2216		Some similarities with McjD protein of Escherichia coli, involved in microcin immunity. Putative secreted membrane protein		0,386	0,368
2_116	EMBL_NAME=PLU2266		Similar to lipase		0,425	0,413
1_265	EMBL_NAME=PLU2468		Some similarities with unknown protein of Photorhabdus		0,457	0,450
6_235	EMBL_NAME=PLU2474		Similar to GTP cyclohydrolase II		0,467	0,487
6_237	EMBL_NAME=PLU2475		Similar to N-terminal region of WD-repeat protein		0,400	0,427
6_239	EMBL_NAME=PLU2476		Similar to unknown protein		0,418	0,453
5_119	EMBL_NAME=PLU2874		Some similarities with tail fiber protein		0,434	0,474
5_117	EMBL_NAME=PLU2875		Similar to putative bacteriophage protein		0,439	0,473
5_115	EMBL_NAME=PLU2876		Similar to putative bacteriophage protein		0,420	0,471
5_302	EMBL_NAME=PLU2877		Similar to putative bacteriophage protein		0,589	0,601
5_113	EMBL_NAME=PLU2878		Similar to putative bacteriophage protein		0,530	0,558

3_127	EMBL_NAME=PLU2879	Similar to putative bacteriophage protein	0,511	0,489
3_125	EMBL_NAME=PLU2881	Similar to putative bacteriophage protein	0,367	0,424
3_121	EMBL_NAME=PLU2887	Similar to bacteriophage protein. Putative transmembrane protein	0,453	0,481
3_119	EMBL_NAME=PLU2891	Similar to unknown bacteriophage protein	0,471	0,519
3_117	EMBL_NAME=PLU2894	Similar to unknown protein	0,496	0,515
3_115	EMBL_NAME=PLU2896	Hypothetical protein	0,368	0,408
1_127	EMBL_NAME=PLU2899	Similar to unknown protein	0,597	0,674
1_125	EMBL_NAME=PLU2900	Similar to unknown protein	0,374	0,381
1_123	EMBL_NAME=PLU2901	Similar to phage terminase large subunit	0,539	0,548
1_121	EMBL_NAME=PLU2903	Unknown protein	0,345	0,394
1_119	EMBL_NAME=PLU2911	Similar to prophage antitermination protein	0,384	0,431
1_117	EMBL_NAME=PLU2916	Some similarities with alpha replication protein of prophage	0,584	0,556
1_115	EMBL_NAME=PLU2917	Similar to bacteriophage protein, putative DNA or RNA helicases	0,460	0,502
1_113	EMBL_NAME=PLU2922	Similar to bacteriophage repressor protein CI	0,468	0,509
4_382	EMBL_NAME=PLU2924	Similar to bacteriophage protein	0,492	0,519
2_370	EMBL_NAME=PLU2931	Similar to bacteriophage protein	0,519	0,560
8_256	EMBL_NAME=PLU2935	Some similarities with bacteriophage recombination protein	0,535	0,571
8_254	EMBL_NAME=PLU2936	Similar to bacteriophage exonuclease	0,413	0,440
12_364	EMBL_NAME=PLU2937	Some similarities with bacteriophage protein	0,419	0,427
12_362	EMBL_NAME=PLU2939	Unknown protein	0,498	0,496
8_252	EMBL_NAME=PLU2940	Some weak similarities with putative histidine kinase	0,435	0,481
8_250	EMBL_NAME=PLU2942	Similar to DNA-methyltransferase	0,469	0,502
8_248	EMBL_NAME=PLU2947	Similar to bacteriophage integrase	0,564	0,587
6_256	EMBL_NAME=PLU2960	Similar to bacteriophage tail fiber protein	0,407	0,445
12_359	EMBL_NAME=PLU3015	Similar to putative glycosyltransferase	0,454	0,419
5_362	EMBL_NAME=PLU3154	Similar to Hcp protein	0,405	0,449
4_90	EMBL_NAME=PLU3238	Similar to putative membrane protein of Y. pestis	0,460	0,976
4_88	EMBL_NAME=PLU3239	Unknown protein	0,555	0,718
4_86	EMBL_NAME=PLU3245	Similar to unknown protein	0,432	0,477
4_84	EMBL_NAME=PLU3246	Similar to vgrG related protein	0,577	0,975
11_341	EMBL_NAME=PLU3249	Similar to unknown protein of Photorhabdus	0,487	1,013
4_82	EMBL_NAME=PLU3250	Highly similar to unknown protein of Photorhabdus	0,512	0,986
2_96	EMBL_NAME=PLU3251	Highly similar to unknown protein of Photorhabdus. Putative secreted protein.	0,395	1,012
2_94	EMBL_NAME=PLU3252	Similar to unknown protein	0,567	1,000
7_362	EMBL_NAME=PLU3284	Highly similar to unknown protein YfhP Escherichia coli	0,655	0,544
9_362	EMBL_NAME=PLU3365	Some similarities with isopentenyl-diphosphate delta-isomerase	0,466	0,342
6_323	EMBL_NAME=PLU3380	Similar to unknown protein	0,680	0,578
5_192	EMBL_NAME=PLU3382	Some similarities with the N-terminal region of tail fiber protein	0,427	0,476
7_178	EMBL_NAME=PLU3383	Similar to unknown protein of Photorhabdus	0,365	0,400
7_180	EMBL_NAME=PLU3384	Weakly similar to unknown bacteriophage protein	0,583	0,562
7_182	EMBL_NAME=PLU3389	Some similarities with unknown protein of Photorhabdus	0,427	0,436
6_329	EMBL_NAME=PLU3390	Hypothetical protein	0,557	0,556
7_184	EMBL_NAME=PLU3391	Some similarities with unknown protein	0,419	0,439
7_186	EMBL_NAME=PLU3392	Some similarities with the central region of structural lytic transglycosylase	0,524	0,543
6_331	EMBL_NAME=PLU3393	Unknown protein	0,576	0,588
7_188	EMBL_NAME=PLU3395	Similar to unknown protein	0,349	0,376
6_333	EMBL_NAME=PLU3397	Hypothetical protein	0,548	0,485
11_360	EMBL_NAME=PLU3398	Weakly similar to unknown protein	0,217	0,240
6_335	EMBL_NAME=PLU3399	Hypothetical protein	0,307	0,313
7_192	EMBL_NAME=PLU3401	Unknown protein	0,518	0,546
9_178	EMBL_NAME=PLU3403	Weakly similar to unknown protein	0,446	0,471
9_180	EMBL_NAME=PLU3404	Weakly similar to unknown protein	0,424	0,441
9_182	EMBL_NAME=PLU3405	Some similarities with conserved hypothetical protein	0,384	0,373
9_184	EMBL_NAME=PLU3406	Similar to putative prophage terminase large subunit	0,533	0,515
9_186	EMBL_NAME=PLU3407	Some similarities with putative prophage terminase small subunit	0,588	0,604
9_188	EMBL_NAME=PLU3411	Unknown protein	0,511	0,529
11_178	EMBL_NAME=PLU3423	Highly similar to tail fiber protein	0,558	0,559
11_180	EMBL_NAME=PLU3424	Highly similar to phage-related baseplate assembly protein	0,438	0,453
8_331	EMBL_NAME=PLU3425	Highly similar to baseplate protein	0,567	0,529
11_182	EMBL_NAME=PLU3426	Similar to tailspike and baseplate protein	0,302	0,312
11_184	EMBL_NAME=PLU3427	Highly similar to hypothetical protein and some similarities with bacteriophage late control gene D protein (GPD)	0,369	0,386
11_186	EMBL_NAME=PLU3428	Similar to unknown protein	0,468	0,472
8_333	EMBL_NAME=PLU3430	Similar to hypothetical protein	0,536	0,497
11_190	EMBL_NAME=PLU3432	Highly similar to tail sheath protein	0,474	0,487
1_376	EMBL_NAME=PLU3435	Weakly similar to mu-like prophage protein	0,520	0,578
8_335	EMBL_NAME=PLU3436	Similar to unknown protein	0,404	0,399
11_192	EMBL_NAME=PLU3437	Similar to unknown protein	0,501	0,519
10_323	EMBL_NAME=PLU3445	Similar to putative membrane protein	0,661	0,594
2_172	EMBL_NAME=PLU3447	Similar to putative soluble lytic murein transglycosylase	0,596	0,619
10_325	EMBL_NAME=PLU3448	Similar to unknown protein	0,650	0,593
2_174	EMBL_NAME=PLU3449	Highly similar to probable DNA adenine methylase of a bacteriophage	0,498	0,525
10_327	EMBL_NAME=PLU3451	Hypothetical gene	0,413	0,384

10_329	EMBL_NAME=PLU3452		Hypothetical protein with two candidate membrane-spanning segments		0,612	0,548
10_331	EMBL_NAME=PLU3453		Some similarities with unknown protein encoded by prophage CP-933T		0,429	0,412
10_333	EMBL_NAME=PLU3455		Similar to unknown protein		0,625	0,573
4_162	EMBL_NAME=PLU3457		Similar to unknown protein		0,400	0,450
4_164	EMBL_NAME=PLU3458		Similar to general secretion pathway protein A		0,348	0,388
10_335	EMBL_NAME=PLU3460		Some similarities with bacteriophage protein Gp16		0,426	0,401
12_325	EMBL_NAME=PLU3464		Some similarities with bacteriophage Q protein		0,528	0,531
12_327	EMBL_NAME=PLU3467		Hypothetical gene		0,535	0,533
4_168	EMBL_NAME=PLU3471		Similar to replicative DNA helicase of Bacteriophage		0,694	0,590
12_331	EMBL_NAME=PLU3475		Some similarities with unknown protein and with YdaT protein of Escherichia coli		0,558	0,537
12_335	EMBL_NAME=PLU3477		Similar to putative repressor protein encoded by cryptic prophage		0,378	0,389
2_339	EMBL_NAME=PLU3479		Hypothetical gene		0,532	0,577
3_376	EMBL_NAME=PLU3483		Similar to bacteriophage protein		0,198	0,226
2_341	EMBL_NAME=PLU3486		Hypothetical gene		0,743	0,590
3_378	EMBL_NAME=PLU3491		Similar to unknown protein YdcQ of Escherichia coli		0,451	0,488
3_382	EMBL_NAME=PLU3504		Unknown protein		0,503	0,549
5_378	EMBL_NAME=PLU3553	fldB		flavodoxin 2	0,418	0,281
4_347	EMBL_NAME=PLU3590		Some similarities with transcriptional regulator		0,257	0,394
5_382	EMBL_NAME=PLU3602		Similar to unknown protein YgfB of Escherichia coli		0,338	0,265
7_376	EMBL_NAME=PLU3646		Similar to unknown protein YacA of Escherichia coli		0,332	0,246
7_378	EMBL_NAME=PLU3663	mraZ		Protein MraZ	0,446	0,464
7_382	EMBL_NAME=PLU3670		Similar to unknown protein YdeN of Bacillus subtilis		0,377	0,392
3_33	EMBL_NAME=PLU3733		Similar to GalR/LacI family transcriptional regulator		0,357	0,405
3_362	EMBL_NAME=PLU3805	ppiB		Peptidyl-prolyl cis-trans isomerase B (PPIase B) (rotamase B)	0,359	0,415
3_275	EMBL_NAME=PLU4240		Hypothetical membrane protein		0,572	0,578
6_38	EMBL_NAME=PLU4241		Highly similar to unknown gene of Photorhabdus luminescens		0,592	0,720
6_62	EMBL_NAME=PLU4320	hsdS		Type I restriction enzyme specificity protein HsdS	0,361	0,395
8_52	EMBL_NAME=PLU4324		Similar to unknown protein		0,521	0,537
8_56	EMBL_NAME=PLU4326		Similar to unknown protein		0,352	0,356
1_279	EMBL_NAME=PLU4327		Hypothetical protein		0,552	0,561
8_58	EMBL_NAME=PLU4328		Similar to unknown protein		0,447	0,482
5_253	EMBL_NAME=PLU4891		Some similarities with O-methyltransferase		0,568	0,548
1_289	EMBL_NAME=PLU4892		Some similarities with O-methyltransferase		0,415	0,470
