# Supplementary material for: Plastic architecture of bacterial genome revealed by comparative genomics of Photorhabdus variants
Source: Genome Biol. 2008 Jul 22;9(7):R117. doi: 10.1186/gb-2008-9-7-r117 (PMC2530875; doi:10.1186/gb-2008-9-7-r117)
Supplement: Additional data file 5 — Presented is a table listing the TT01/I amplified genes in TT01α/I and VAR* variants, according to whole-genome comparison using DNA microarray. [file gb-2008-9-7-r117-S5.pdf]

Additional data file 5. TT01/I genes amplified in VAR* variant (yellow lines) and in both TT01α/I and VAR* variants (blue lines) according whole-genome comparison using DNA micro-array.

Microarray position	plu	Name	Similarity	Protein	Ratio var/wt	Ratio mut/wt
7_71	EMBL_NAME=PLU0213	envZ		osmolarity sensor protein	1,472	0,996
5_352	EMBL_NAME=PLU0595		Some similarities with the C-terminal region of transposases		1,419	1,148
10_141	EMBL_NAME=PLU0634		Similar to toxin secretion ATP-binding protein		1,589	1,592
11_140	EMBL_NAME=PLU0769	mrfA	Highly similar to major fimbrial subunit polypeptide.	MrfA protein	1,651	1,097
11_138	EMBL_NAME=PLU0771	mrfC		Outer membrane usher protein MrfC	1,671	1,044
11_136	EMBL_NAME=PLU0772	mrfD	Highly similar to periplasmic fimbrial chaperone precursor	MrfD protein	1,566	1,039
6_271	EMBL_NAME=PLU0775	mrfF	Similar to fimbrial protein	MrfF protein	1,609	1,081
6_269	EMBL_NAME=PLU0776	mrfG	Highly similar to fimbrial protein	MrfG protein	1,669	1,078
11_132	EMBL_NAME=PLU0777	mrfH	Highly similar to fimbrial protein	MrfH protein	1,696	1,054
1_338	EMBL_NAME=PLU0778	mrfJ		MrfJ protein	1,586	1,115
6_267	EMBL_NAME=PLU0779		Similar to putative fimbrial-like protein		1,506	1,019
11_130	EMBL_NAME=PLU0780		Similar to putative fimbrial chaperone		1,665	1,073
9_140	EMBL_NAME=PLU0785		Highly similar to unknown protein of Photorhabdus. Probable transmembrane protein with 3 candidate membrane-spanning segments.		1,538	1,065
6_265	EMBL_NAME=PLU0786		Some similarities with unknown protein of Photorhabdus. Probable transmembrane protein with 3 candidate membrane-spanning segments.		1,534	1,079
9_138	EMBL_NAME=PLU0787		Similar to putative fimbrial chaperone		1,658	1,091
6_263	EMBL_NAME=PLU0788		Highly similar to unknown protein of Photorhabdus. Hypothetical secreted protein		1,709	1,050
9_134	EMBL_NAME=PLU0790		Weakly similar to putative fimbrial chaperone		1,585	1,049
9_132	EMBL_NAME=PLU0791		Similar to outer membrane usher protein precursor		1,529	1,040
9_130	EMBL_NAME=PLU0792		Highly similar to unknown protein of Photorhabdus. Probable transmembrane protein with 2 candidate membrane-spanning segments.		1,542	1,072
7_144	EMBL_NAME=PLU0794		Similar to adenylosuccinate synthetase		1,573	1,073
7_142	EMBL_NAME=PLU0795		Similar to phosphoglycolate phosphatase		1,563	1,037
7_136	EMBL_NAME=PLU0798		Similar to efflux pump		1,566	1,073
7_134	EMBL_NAME=PLU0799	tnaA		Tryptophanase (L-tryptophan indole-lyase) (TNase)	1,596	1,043
7_132	EMBL_NAME=PLU0800	mtr		Tryptophan-specific transport protein (Tryptophan permease)	1,461	1,034
7_130	EMBL_NAME=PLU0801		Similar to unknown protein		1,552	1,066
5_142	EMBL_NAME=PLU0803		Highly similar to putative serine-threonine dehydratase		1,421	1,029
5_138	EMBL_NAME=PLU0805	tccA3		Insecticidal toxin complex protein TtcA	1,643	1,036
5_136	EMBL_NAME=PLU0806	tccB3		Insecticidal toxin complex protein TtcB	1,445	1,051
11_334	EMBL_NAME=PLU0807		Weakly similar to hypothetical protein		1,668	1,084
11_332	EMBL_NAME=PLU0808		Similar to unknown protein		1,552	1,056
11_330	EMBL_NAME=PLU0809		Hypothetical gene		1,632	1,094
5_132	EMBL_NAME=PLU0811	afuB		Permease component of transport system for ferric iron, AfuB protein	1,460	0,995
3_144	EMBL_NAME=PLU0813	uhpC		Regulatory protein UhpC	1,495	1,075
3_138	EMBL_NAME=PLU0820		Highly similar to unknown protein of Photorhabdus and some similarities with gp30 protein of Bacteriophage		1,409	1,054
11_326	EMBL_NAME=PLU0827		Hypothetical gene		1,575	1,110
3_132	EMBL_NAME=PLU0830		Weakly similar to phospholipase A1		1,425	1,060
3_130	EMBL_NAME=PLU0831	ampC		beta-lactamase class C (cephalosporinase)	1,404	1,070
6_259	EMBL_NAME=PLU0835	agaV	Highly similar to PTS system, cytoplasmic, N-acetylgalactosamine-specific	PTS system, N-acetylgalactosamine-specific IIB component 2 (EIIB-AGA) (N-acetylgalactosamine-perme	1,652	1,039
12_159	EMBL_NAME=PLU0843	speE		spermidine synthase (putrescine aminopropyltransferase)	1,400	1,053
9_336	EMBL_NAME=PLU0844		Similar to unknown protein YacC of Escherichia coli		1,465	1,089
12_157	EMBL_NAME=PLU0845	cueO		Blue copper oxidase CueO precursor	1,498	1,042
9_334	EMBL_NAME=PLU0846		Hypothetical gene		1,521	1,062
12_155	EMBL_NAME=PLU0847		Hypothetical transmembrane protein		1,455	1,004
4_271	EMBL_NAME=PLU0848		Weakly similar to ATP-dependent Clp protease ATP-binding subunit ClpX		2,204	1,496
9_332	EMBL_NAME=PLU0850		Weakly similar to putative integrase		1,438	1,060
12_153	EMBL_NAME=PLU0852		Highly similar to toxin secretion ABC transporter ATP-binding protein.		1,484	1,027
9_330	EMBL_NAME=PLU0855		Similar to N-terminal region of colicin V secretion protein		1,734	1,031
12_149	EMBL_NAME=PLU0856		Similar to unknown protein		1,578	1,036
9_328	EMBL_NAME=PLU0857		Weakly similar to different AG rich proteins		1,538	1,029
4_267	EMBL_NAME=PLU0860		Highly similar to hypoxanthine phosphoribosyltransferase of Escherichia coli		1,659	0,989
4_265	EMBL_NAME=PLU0862		Similar to unknown protein		1,506	1,045
9_324	EMBL_NAME=PLU0865		Similar to virulence-associated protein and proteic killer active protein		1,605	1,125
9_322	EMBL_NAME=PLU0866		Highly similar to proteic killer suppression protein		1,473	1,071
12_147	EMBL_NAME=PLU0867		Highly similar to putative carbonic anhydrase YadF of Escherichia coli		1,510	1,044
12_145	EMBL_NAME=PLU0868	yadG		ABC transporter ATP-binding protein YadG	1,507	1,034
10_159	EMBL_NAME=PLU0869	yadH		ABC transporter, integral membrane protein YadH	1,494	1,061
10_155	EMBL_NAME=PLU0872	panB		3-methyl-2-oxobutanoate hydroxymethyltransferase	1,408	1,058
4_261	EMBL_NAME=PLU0873	folK		7,8-dihydro-6-hydroxymethylpterin-pyrophosphokinase	1,421	1,055
10_153	EMBL_NAME=PLU0874	pcnB		Poly(A) polymerase (PAP) (Plasmid copy number protein)	1,453	1,040
10_151	EMBL_NAME=PLU0875		Similar to unknown protein YadB of Escherichia coli		1,468	1,056
4_259	EMBL_NAME=PLU0876	dksA		DnaK suppressor protein	1,410	1,064
10_149	EMBL_NAME=PLU0877	sfsA		Sugar fermentation stimulation protein A	1,500	1,051
5_65	EMBL_NAME=PLU0882	hrpB		ATP-dependent helicase HrpB	1,558	1,036
3_79	EMBL_NAME=PLU0883	mrcB		penicillin-binding protein 1B	1,609	1,080
3_77	EMBL_NAME=PLU0884		Some similarities with killer protein of pyocin S3 and highly similar to Photorhabdus luminescens unknown protein		1,480	1,051
2_271	EMBL_NAME=PLU0886		Similar to pyocin S3 immunity protein		1,603	1,078
2_269	EMBL_NAME=PLU0887		Similar to C-terminal region of klebicin B, pyocin S2 and the killer protein of pyocin S1		1,559	1,036
7_334	EMBL_NAME=PLU0888		Similar to colicin / pyocin immunity protein		1,489	1,085
7_332	EMBL_NAME=PLU0892		Similar to colicin / pyocin immunity protein		1,496	1,068

7_330	EMBL_NAME=PLU0894		Similar to pyocin S3 immunity protein		1,424	1,085
7_328	EMBL_NAME=PLU0895		Some similarities with transcriptional regulator, Cro/CI family		1,627	1,097
3_75	EMBL_NAME=PLU0896		Similar to unknown protein		1,645	1,108
3_73	EMBL_NAME=PLU0897		Similar to peptide synthetases, pyoverdine synthetase and antibiotics synthetases		1,451	1,075
3_71	EMBL_NAME=PLU0898		Some similarities with peptide synthetase like pristinamycin I synthase 3, actinomycin synthetase III and virginiamycin S synthetase. Putative transmembrane protein		1,426	1,076
3_69	EMBL_NAME=PLU0899		Some similarities with peptide synthetase like pristinamycin I synthase 3, actinomycin synthetase III and virginiamycin S synthetase. Putative transmembrane protein		1,434	1,009
7_326	EMBL_NAME=PLU0900		Similar to protein gp48 from phage N15		1,471	1,092
7_324	EMBL_NAME=PLU0901		Similar to virulence plasmid protein		1,645	1,075
7_322	EMBL_NAME=PLU0904		Highly similar to unknown protein YadR of Escherichia coli		1,548	1,040
1_71	EMBL_NAME=PLU0909	rumA		23S rRNA (Uracil-5-)-methyltransferase rumA (23S rRNA(M-5-U1939)-methyltransferase)	1,488	1,037
1_69	EMBL_NAME=PLU0910	relA		GTP pyrophosphokinase	1,525	1,048
11_383	EMBL_NAME=PLU0913	eno		Enolase (2-phosphoglycerate dehydratase) (2-phospho-D-glycerate hydro-lyase)	1,585	1,072
11_381	EMBL_NAME=PLU0914		Similar to hypothetical phage protein		1,629	1,111
11_379	EMBL_NAME=PLU0915		Similar to cysteine desulfurase (NifS protein homolog)		1,669	1,094
11_377	EMBL_NAME=PLU0916		Some similarities with transporter protein		1,695	1,068
11_375	EMBL_NAME=PLU0926		Similar to sodium:sulfate symporter-family protein		1,726	1,095
9_383	EMBL_NAME=PLU0933		Hypothetical protein		1,742	1,071
9_381	EMBL_NAME=PLU0934		Weakly similar to Inositol-1-monophosphatase (IMPase), SuhB protein of Escherichia coli		1,696	1,047
9_379	EMBL_NAME=PLU0935		Weakly similar to unknown protein		1,816	1,085
9_377	EMBL_NAME=PLU0937		Some similarities with transporter		1,631	1,097
9_375	EMBL_NAME=PLU0938		Highly similar to unknown protein of Photorhabdus and some weak similarities with oxidase.		1,460	1,046
9_373	EMBL_NAME=PLU0939	mltD		membrane-bound lytic murein transglycosylase D precursor (murein hydrolase D)	1,557	1,102
9_371	EMBL_NAME=PLU0940	gloB		hydroxyacylglutathione hydrolase (glyoxalase II)	1,518	1,118
7_383	EMBL_NAME=PLU0943	dnaQ		DNA polymerase III epsilon chain	1,671	1,069
5_334	EMBL_NAME=PLU0944		Similar to sugar fermentation stimulation protein B (Ner-like protein) of Escherichia coli (Pln protein of Photorhabdus luminescens)		1,439	1,028
7_381	EMBL_NAME=PLU0945		Weakly similar to transcriptional regulator, LuxR family		1,498	1,053
7_379	EMBL_NAME=PLU0946	tktA		transketolase 1	1,539	1,010
5_332	EMBL_NAME=PLU0947		Similar to unknown protein		1,527	1,133
7_377	EMBL_NAME=PLU0949		Similar to transcriptional regulator		1,400	1,058
7_375	EMBL_NAME=PLU0950		Some similarities with eugenol hydroxylase flavoprotein subunit		1,608	1,081
5_330	EMBL_NAME=PLU0952		Some similarities with unknown protein		1,556	1,081
5_328	EMBL_NAME=PLU0954		Weakly similar to probable short-chain dehydrogenase		1,421	1,074
7_373	EMBL_NAME=PLU0955	epd		D-erythrose 4-phosphate dehydrogenase	1,566	1,087
7_371	EMBL_NAME=PLU0956	pgk		phosphoglycerate kinase	1,613	1,095
5_326	EMBL_NAME=PLU0959		Similar to unknown protein		1,476	1,082
5_383	EMBL_NAME=PLU0960	tccC2		Insecticidal toxin complex protein TtfC	1,571	1,041
5_381	EMBL_NAME=PLU0961	tcdB1	Highly similar to insecticidal toxin complex protein TcdB	Insecticidal toxin complex protein TtfE	1,624	1,099
5_379	EMBL_NAME=PLU0962	tcdA1	Highly similar to insecticidal toxin complex protein TcdA (toxin A)	Insecticidal toxin complex protein TtfD	1,646	1,117
5_377	EMBL_NAME=PLU0963	tciR2	Similar to LysR-type transcriptional activator		1,652	1,042
5_375	EMBL_NAME=PLU0964	tccC5		Insecticidal toxin complex protein TteC	1,410	1,049
5_373	EMBL_NAME=PLU0965	tcdA4		Insecticidal toxin complex protein TteD	1,580	1,038
5_324	EMBL_NAME=PLU0968	tchA	Weakly similar to putative holin protein of prophage CP-933X		1,580	1,117
3_381	EMBL_NAME=PLU0970	tcdB2		Insecticidal toxin complex protein TtdD2	1,552	1,071
3_379	EMBL_NAME=PLU0971	tcdA2		Insecticidal toxin complex protein TtdD1	1,510	1,125
3_375	EMBL_NAME=PLU0973		Highly similar to putative di-tripeptide transporter protein YhiP of Escherichia coli		1,472	1,059
5_322	EMBL_NAME=PLU0978		Hypothetical gene		1,591	1,052
1_383	EMBL_NAME=PLU0980	hpaA		regulator of the 4HPA-hydroxylase operon	1,450	1,076
7_384	EMBL_NAME=PLU1104		Similar to lactoylglutathione lyase and related lyases		1,712	1,062
5_121	EMBL_NAME=PLU2873		Similar to bacteriophage tail fiber protein		1,910	2,106
7_368	EMBL_NAME=PLU3343	rseC		Sigma-E factor regulatory protein RseC	1,832	1,131
9_243	EMBL_NAME=PLU3631		Similar to probable fimbrial chaperone YcbR precursor of Escherichia coli		1,472	1,019
9_245	EMBL_NAME=PLU3634		Similar to the N-terminal region of outer membrane usher protein precursor		1,551	1,013
2_46	EMBL_NAME=PLU4221		Unknown protein		1,689	1,173
2_48	EMBL_NAME=PLU4222		Similar to vgrG related protein		2,615	1,421
