# Supplementary material for: Plastic architecture of bacterial genome revealed by comparative genomics of Photorhabdus variants
Source: Genome Biol. 2008 Jul 22;9(7):R117. doi: 10.1186/gb-2008-9-7-r117 (PMC2530875; doi:10.1186/gb-2008-9-7-r117)
Supplement: Additional data file 6 — Presented is a table listing strains and plasmids used in this study. [file gb-2008-9-7-r117-S6.pdf]

Additional data file 6. Strains and plasmids used in this study

Strain or plasmid	Relevant Genotype or characteristics	Source
Strain		
Photorhabdus		
TT01$_{/I}$	Wild type, primary variant	CIP105565
TT01$_{/II}$	Secondary variant derived from TT01$_{/I}$	[30]
TT01α$_{/I}$	Genomic variant derived from TT01$_{/I}$	[84]
TT01α$_{/II}$	Secondary variant derived from TT01α$_{/I}$	[84]
TT01α'$_{/II}$	Secondary variant derived from TT01α$_{/I}$	This study
VAR	Secondary variant<br>Sub-clonal population of TT01α'$_{/II}$	This study
REV	Primary variant or revertant<br>Sub-clonal population of TT01α'$_{/II}$	This study
INT	Intermediate variant<br>Sub-clonal population of TT01α'$_{/II}$	This study
VAR*	Stabilized secondary variant derived from VAR	This study
Escherichia coli		
XL1Blue	F' *proAB lacI*^{q}ZΔM15 Tn*10*(Tet^{R})	Laboratory stock
Plasmid		
pUC19	Ap^{R} cloning vector	Laboratory stock
plg2711	pcDNA2.1 harboring a fragment corresponding to coordinates 3825627 to 3835803 of the TT01$_{/I}$ genome	[42]
plbac4g08	pBeloBAC harbouring a fragment corresponding to coordinates 892690 to 942633 of the TT01$_{/I}$ genome	[42]
plbac6h12	pBeloBAC harbouring a fragment corresponding to coordinates 942372 to 993628 of the TT01$_{/I}$ genome	[42]
plbac3a10	pBeloBAC harbouring a fragment corresponding to coordinates 1008219 to 1070139 of the TT01$_{/I}$ genome	[42]
plbac3c04	pBeloBAC harbouring a fragment corresponding to coordinates 1069964 to 1126366 of the TT01$_{/I}$ genome	[42]
plbac2f12	pBeloBAC harbouring a fragment corresponding to coordinates 1126246 to 1157150 of the TT01$_{/I}$ genome	[42]
