# Supplementary material for: Plastic architecture of bacterial genome revealed by comparative genomics of Photorhabdus variants
Source: Genome Biol. 2008 Jul 22;9(7):R117. doi: 10.1186/gb-2008-9-7-r117 (PMC2530875; doi:10.1186/gb-2008-9-7-r117)
Supplement: Additional data file 7 — Presented is a table listing primers used in this study. [file gb-2008-9-7-r117-S7.pdf]

Additional data file 7: Primers used in this study.

**Primer name**	**Primer sequence**
P*lopT1*.rev	TCATTGTGTTTTAGGCTCGT
P*lopT1*.fw	GGGCTGTATTTTAGAAACAG
R-3236	AATATCCTGAAAAGAAGGGC
F-3249	GGGTTCGATG TGATTAGTTT
R-3238bis	ATGCTACACA TAGCCCCATC
F-3254	GATTCCCGCTTATTTATGG
L-1954	GGAGAAGTAGTTAAACAGGTTGACG
R-1954	ACCCTCTTTAGTAATAGTGGCTTCG
L-0778	TAGGCATTAGTCAGCAACAACTCTC
R-0778	CTTCCGCAATAGGGATATATTGGTC
L-0943	TATATTCAGCCGGATAGATTGGTC
R- 0943	GCTATCAGTAATCTTGCAGAAATCC
L-1051	TACCAGTAGTAATGCCAGTGTTGC
R-1051	TCAACATATTCTCGACTGTCTTACG
L-0004	ATACACGAAGAAGAAGGTGTTTCAG
R-0004	TACCTGTCTGTTCAGTTTCTCCAAC
*Pst*IdMutF	GCGCCTGCAGCCCTGAATCTGACCGGATAA
*Xba*IdMutR	GCGCTCTAGATTTCGAAGGGCTGATAATGG
27f	GTGCTGCAGAGAGTTTGATCCTGGCTCAG
1492r	CACGGATCCTACGGGTACCTTGTTACGACTT
